# Pan-cancer atlas of somatic core and linker histone mutations

**DOI:** 10.1038/s41525-023-00367-8

**Published:** 2023-08-28

**Authors:** Erin R. Bonner, Adam Dawood, Heather Gordish-Dressman, Augustine Eze, Surajit Bhattacharya, Sridevi Yadavilli, Sabine Mueller, Sebastian M. Waszak, Javad Nazarian

**Affiliations:** 1 Center for Genetic Medicine Research, Children’s National Hospital, Washington, DC USA; 2 Department of Biostatistics, Children’s National Hospital, Washington, DC USA; 3 Department of Neurology, Neurosurgery and Pediatrics, University of California San Francisco, San Francisco, CA USA; 4 Department of Oncology, University Children’s Hospital Zürich, Zürich, Switzerland; 5 Laboratory of Computational Neuro-Oncology, Swiss Institute for Experimental Cancer Research (ISREC), School of Life Sciences, École Polytechnique Fédérale de Lausanne (EPFL), Lausanne, Switzerland; 6 Centre for Molecular Medicine Norway (NCMM), Nordic EMBL Partnership, University of Oslo and Oslo University Hospital, Oslo, Norway; 7 Department of Neurology, University of California, San Francisco, San Francisco, CA USA

**Keywords:** Cancer genetics, Paediatric cancer, Cancer genomics

## Abstract

Recent genomic data points to a growing role for somatic mutations altering core histone and linker histone-encoding genes in cancer. However, the prevalence and the clinical and biological implications of histone gene mutations in malignant tumors remain incompletely defined. To address these knowledge gaps, we analyzed somatic mutations in 88 linker and core histone genes across 12,743 tumors from pediatric, adolescent and young adult (AYA), and adult cancer patients. We established a pan-cancer histone mutation atlas contextualized by patient age, survival outcome, and tumor location. Overall, 11% of tumors harbored somatic histone mutations, with the highest rates observed among chondrosarcoma (67%), pediatric high-grade glioma (pHGG, >60%), and lymphoma (>30%). Previously unreported histone mutations were discovered in pHGG and other pediatric brain tumors, extending the spectrum of histone gene alterations associated with these cancers. Histone mutation status predicted patient survival outcome in tumor entities including adrenocortical carcinoma. Recurrent pan-cancer histone mutation hotspots were defined and shown to converge on evolutionarily conserved and functional residues. Moreover, we studied histone gene mutations in 1700 pan-cancer cell lines to validate the prevalence and spectrum of histone mutations seen in primary tumors and derived histone-associated drug response profiles, revealing candidate drugs targeting histone mutant cancer cells. This study presents the first-of-its-kind atlas of both core and linker histone mutations across pediatric, AYA, and adult cancers, providing a framework by which specific cancers may be redefined in the context of histone and chromatin alterations.

## Introduction

Somatic mutations altering histone-encoding genes are emerging as tumorigenic drivers in pediatric and adult cancers. Most of these mutations alter core histone-encoding genes (H2A, H2B, H3, H4). Missense mutations altering histone tail residues of H3 proteins are the most well-characterized histone mutations: H3 K27M in pediatric diffuse midline glioma (DMG, >80% of cases)^1–4^; H3 G34R/V in hemispheric pediatric high-grade glioma (pHGG, 15–25%)^4^; H3 K36M in chondrosarcoma (95%) and H3 G34W/L in giant cell tumors of bone (92%)^5^. These hotspot H3 mutations and more recently identified oncohistones (e.g., H2B E53D, H2B E76K; H3 E97K) lead to global epigenome alterations^1,2,6–11^ and/or perturb nucleosome structure and stability^12–16^.

While there is an established role for core histone mutations in several cancers, the prevalence of and role for linker histone (H1) alterations across cancers remains incompletely defined. H1 histones bind to linker DNA spanning adjacent nucleosomes, facilitate chromatin compaction and higher order chromatin structure, and regulate epigenetic patterning^17–19^. H1 mutations have emerged as key genomic alterations in lymphomas, resulting in chromatin de-compaction and aberrant expression of developmentally regulated genes^17,20,21^.

Collectively, the growing body of literature points to increasing evidence for both core and linker histone gene mutations in cancer. However, there has yet to be a comprehensive pan-cancer interrogation of the full landscape of somatic mutations affecting histone-encoding genes from pediatric to adult cancers. To address this knowledge gap, we created a detailed pan-cancer histone mutational atlas by analyzing publicly available whole genome/exome data from three large-scale studies (The Cancer Genome Atlas, Pan Cancer Analysis of Whole Genomes, and Pediatric Brain Tumor Atlas) representing a total of 12,743 cancer genomes from >30 solid and hematological malignancies. We further validated patterns observed in primary tumors using cancer cell lines from the Cancer Dependency Map (DepMap) Project to delineate the effects of histone mutations on drug response profiles.

## Results

### Integration and mapping of histone mutations across 12,743 cancer genomes

We generated a comprehensive atlas of core and linker histone gene mutations in cancer by interrogating publicly available whole genome/exome sequencing data from three studies^1^: The Cancer Genome Atlas (TCGA) (*n* = 10,131 subjects)^22^, (2) the International Cancer Genome Consortium Pan-Cancer Analysis of Whole Genomes (ICGC PCAWG) (*n* = 1798 subjects)^23^, and (3) the Pediatric Brain Tumor Atlas (PBTA) (*n* = 814 subjects)^24^ (Table 1). In total, we analyzed paired tumor-germline genomes from 12,743 subjects representing >30 solid and hematological malignancies for all age groups (pediatric: 0–14 years, *n* = 856; adolescent/young adult (AYA): 15–39 years, *n* = 1302; adult: 40+ years, *n* = 10,441; age NA, *n* = 144). Nonsynonymous mutations were queried across 88 histone protein-encoding genes (*n* = 10 linker, 78 core), representing the most updated list of histone genes from the HUGO Gene Nomenclature Committee (HGNC) at the time of analysis.

**Table 1:** Patient cohort for analysis.

Patient cohort for analysis			
Study	TCGA	ICGC PCAWG	PBTA
Release	MC3	Consensus SNV/indel release	r18
*n*	10,131	1798	814
Ages	Pediatric (*n* = 3) AYA (*n* = 1057) Adult (*n* = 9022) NA = 49	Pediatric (*n* = 161) AYA (*n* = 123) Adult (*n* = 1419) NA = 95	Pediatric (*n* = 692) AYA (*n* = 122)
Cancers	CNS (GBM, LGG), other solid and hematological (AML, BNHL) cancers (>30 types)	CNS (LGG, medulloblastoma), other solid and hematological (AML, BNHL) cancers (>20 types)	CNS cancers (>50 sub-types)

A total of 12,743 subjects were included from three studies: The Cancer Genome Atlas (TCGA) release MC3, International Cancer Genome Consortium Pan Cancer Analysis of Whole Genomes (ICGC PCAWG) consensus SNV/indel release, and Pediatric Brain Tumor Atlas (PBTA) version r18. More than 33 cancer types were represented across all ages (pediatric, adolescent/young adult (AYA), and adult).

*n* number of subjects, *NA* age not available, *CNS* central nervous system, *GBM* glioblastoma multiforme *LGG* low-grade glioma, *AML* acute myeloid leukemia, *BNHL* B cell non-Hodgkin’s lymphoma.

### Atlas of core and linker histone mutations in pediatric, AYA, and adult cancers

**Fig. 1:**
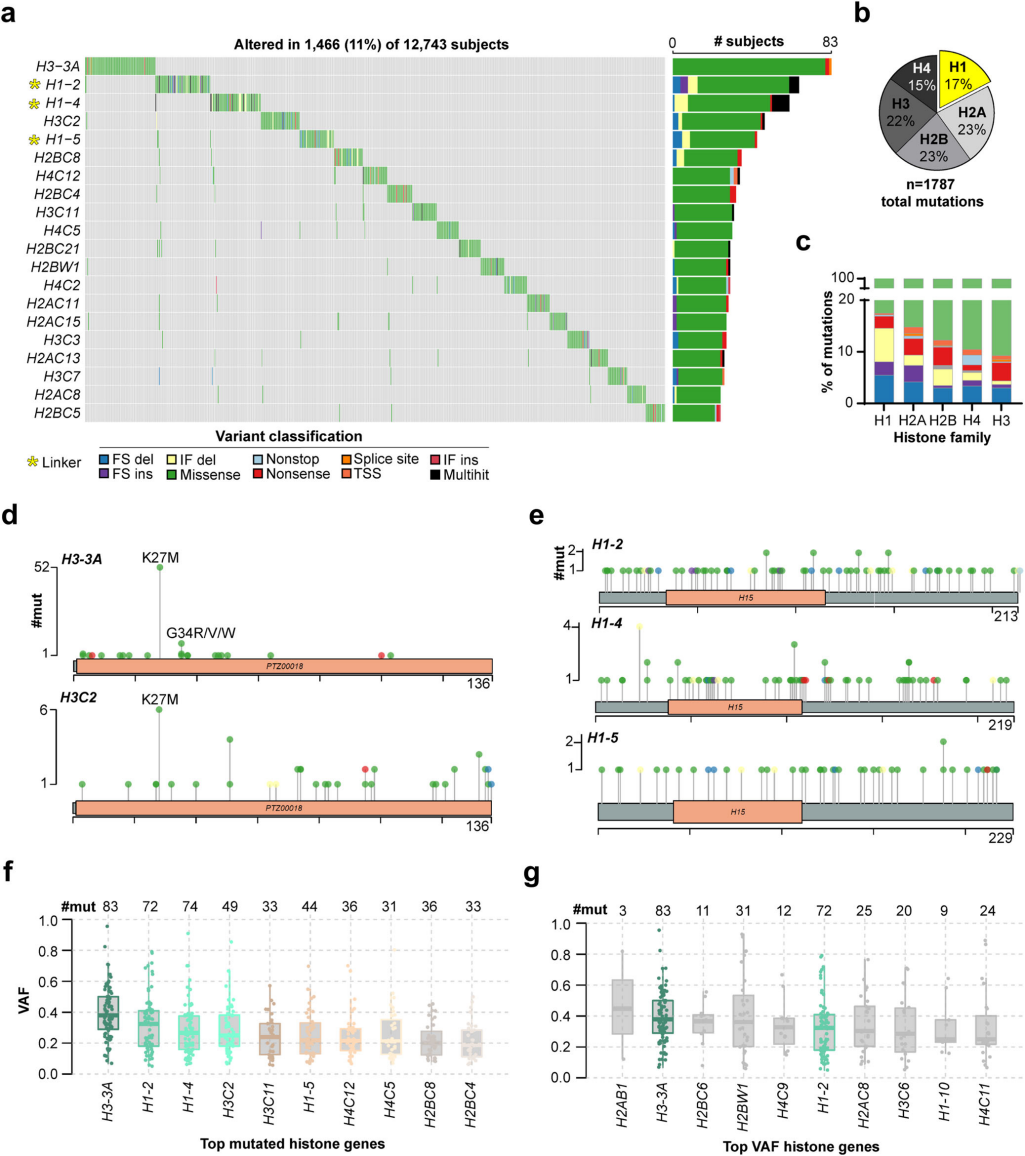
Pan-cancer genomic landscape of core and linker histone mutations. **a** Oncoplot showing the top 20 most recurrently mutated histone genes across cancers. Linker histone genes are denoted with a yellow asterisk. **b** Distribution of histone mutation events (*n* = 1787) across histone families. **c** Mutation types affecting genes of each histone family. Y-axis shows the percentage of each mutation type (variant classification, color key in **a**) out of the total mutations affecting that histone family. **d** Lollipop plots showing mutations affecting the topmost recurrently mutated core histone genes *H3-3A* and *H3C2*. **e** Lollipop plots showing mutations affecting the topmost mutated H1 genes *H1-2/4/5*. Boxplots showing VAF distributions of the topmost recurrently mutated histone genes (**f**) and of histone genes with the highest average VAFs (**g**). Boxplots show the upper and lower quartiles and the median VAF. *H3-3A* and *H1-2* are shown in color in **g** to highlight that they are both the most mutated, and have the highest average VAFs, of all histone genes. Abbreviations: FS frameshift, del deletion, ins insertion, TSS transcription start site, IF in frame, VAF variant allele frequency.

Histone protein-altering somatic mutations were identified in a striking 11.5% of subjects (*n* = 1466), with 1787 mutation events affecting 80 histone-encoding genes (Fig. 1a, Supplementary Data File 1). Core histone mutations were distributed evenly between the four core histone families, while H1 mutations comprised 17% of all mutation events (Fig. 1b). H1 genes exhibited the highest percentage of putative loss-of-protein-function mutations (e.g., disruptive frameshifts and truncating mutations) out of all histone families (Fig. 1c). The topmost recurrently mutated histone genes at pan-cancer level were H3-encoding genes *H3-3A* and *H3C2*, and H1-encoding genes *H1-2/4/5* (Fig. 1a). When accounting for gene length, *H3-3A* and *H3C2* were still amongst the top ten histone genes with the highest mutation rates (mutation rate = log_10_(*n* + 1)/CDS, with *n* = mutation count and CDS = coding sequence length), whereas H1 genes were not, suggesting that the high H1 mutation count could be partially attributed to the longer length of these genes. Most (76%) *H3-3A* mutations were H3 K27M (*n* = 52/83, 63%) and H3 G34R/V/W mutations (*n* = 11/83, 13%; Fig. 1d). These hotspot events accounted for only 12% of *H3C2* mutations (H3 K27M, *n* = 6/49). Among H1 genes, both missense and disruptive/frameshift mutations were distributed broadly across the globular and C-terminal domains of the protein rather than concentrated in a specific region (Fig. 1e). This pan-cancer mutational pattern aligns with previous reports of H1 mutations in lymphomas^20,21^.

### Clonality of histone gene mutations identifies putative driver histone genes

We queried mutation VAF across histone genes to identify putative clonal driver genes with somatic VAFs near 50%, consistent with clonal cancer mutations (assuming diploid genomes and high tumor purity^25^). VAFs across all mutated histone genes ranged between 14–46% (average = 26%, median = 25%). There were no significant differences in average VAF (VAF_avg_) between core versus linker histone genes (Mann–Whitney test, *p* = 0.18, Supplementary Fig. 1A), or between each individual histone family (H1, H2A, H2B, H3, H4; Supplementary Fig. 1B), indicating that clonality was gene-specific, rather than histone family-specific. Among histone genes exhibiting the highest mutation burdens (i.e., top 10 most mutated histone genes, Fig. 1f), *H3–3A* and *H1–2* also exhibited the highest VAF_avg_ (30–40%), indicating clonality of mutations affecting these genes. We also identified genes with the highest VAF_avg_ across all histone genes, which again included the known oncogene *H3-3A* as well as *H1-2*, highlighting *H1-2* as a potential driver gene (Fig. 1g). When considering clonality at both the gene and the mutation level, there were several established oncohistone mutations with VAFs above 30–40%, including *H3-3A* K27M (*n* = 52, VAF_avg_ = 43%), *H3C2* K27M (*n* = 6, VAF_avg_ = 46%), *H3-3A* G34R (*n* = 8, VAF_avg_ = 43%), and *H3-3B* K36M (*n* = 6, VAF_avg_ = 32%). H1 mutations also emerged as clonal and recurrent in at least two patients, including *H1-4* L42V (*n* = 2, VAF_avg_ = 40%), *H1-5* K187N (*n* = 2, VAF_avg_ = 34%), and *H1-2* P146S (*n* = 2, VAF_avg_ = 31%).

The relatively lower VAF_avg_ of mutations affecting other histone genes suggested that, with some exceptions (Fig. 1g), most histone mutations were potentially sub-clonal events. This finding aligns with studies of recently reported oncohistones (e.g., nucleosome destabilizing H2B E76K, which similarly occurred at a VAF consistent with sub-clonality, approx. 20%) suggesting that these events may increase cancer development and/or progression without being the primary driver mutation^12^.

### Spectrum and prevalence of core and linker histone mutation rates across cancer types and ages

**Fig. 2:**
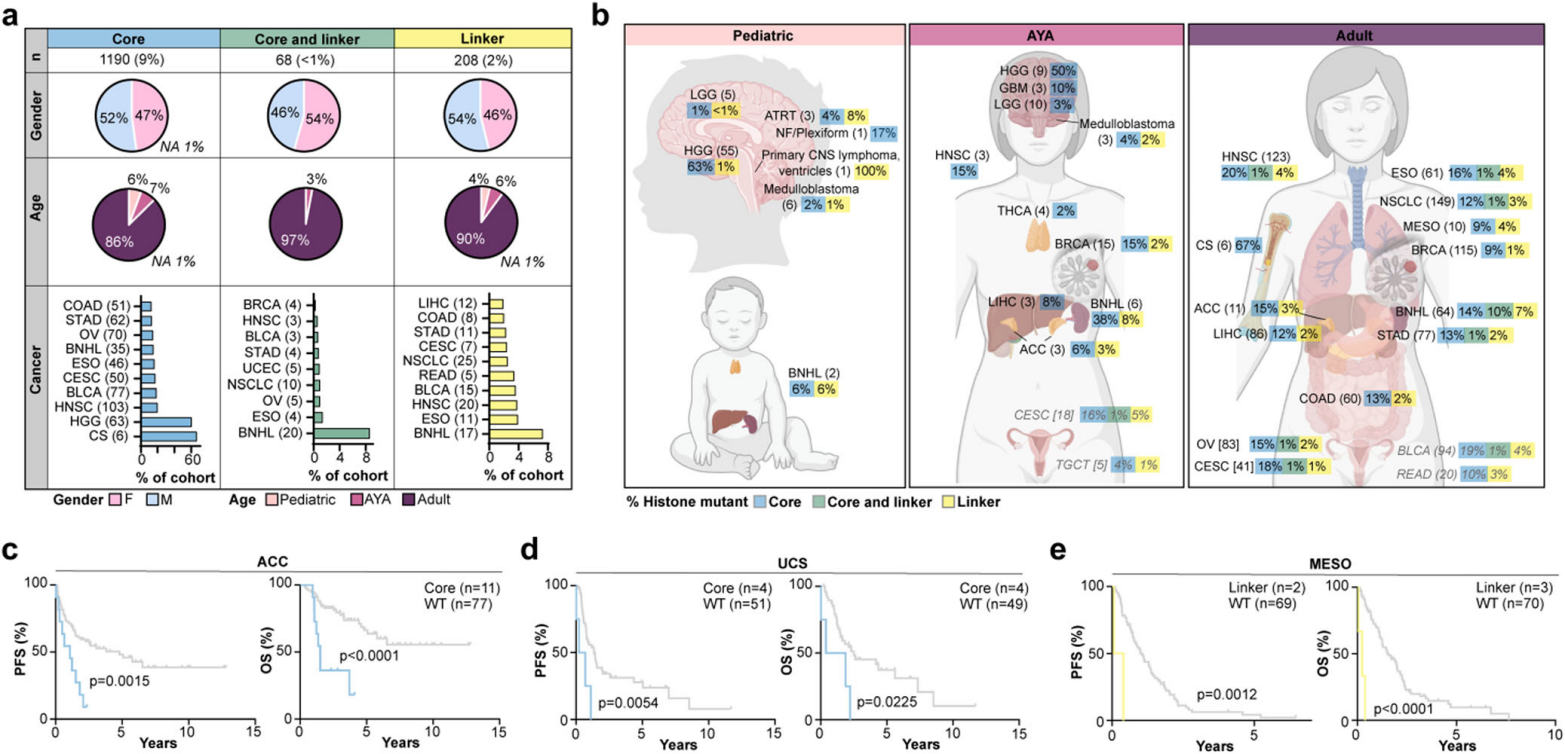
Histone mutation rates and clinical implications across cancers. **a** Summary characteristics of subjects harboring core, co-occurring core and linker, or linker histone mutations. The number and percentage of affected cases (out of *n* = 12,743 total; *top row*), sex and age distribution (*rows 2-3*), and the most affected cancer types (*bottom row*) are shown. For most affected cancer types, only those with ≥5 (core; linker) or ≥3 (co-occurring core and linker) mutant subjects are shown. **b** Histone mutation rates across age groups (pediatric, AYA, adult). Bracketed numbers = number of mutant subjects. Percent of mutant subjects from each cohort is indicated in colored boxes (core = blue, co-occurring core and linker mutant = green, linker = yellow). AYA and adults: the top 15 cancers with the highest mutation rates, and with ≥5 mutant subjects, are shown (except for AYA medulloblastoma, 3 mutant subjects). Gray italics: tissue representing the cancer type is not shown in the diagram. Significantly shorter PFS (*left*) and OS (*right*) outcomes in core histone mutant adrenocortical carcinoma (**c**) and uterine carcinosarcoma (**d**) relative to histone WT subjects, and among linker histone mutant mesothelioma (**e**) relative to histone wildtype mesothelioma subjects. *P*-values were determined by log-rank (Mantel–Cox) tests. Abbreviations: pHGG pediatric high-grade glioma (hem. hemispheric, mid. midline), LIHC liver hepatocellular carcinoma, COAD colon adenocarcinoma, STAD stomach adenocarcinoma, CESC cervical squamous cell carcinoma and endocervical adenocarcinoma, NSCLC non-small cell lung cancer, UCEC uterine corpus endometrial carcinoma, NF neurofibroma, READ rectum adenocarcinoma, BLCA urothelial bladder carcinoma, HNSC head and neck squamous cell carcinoma, ESO esophageal carcinoma, BNHL B cell non-Hodgkin’s lymphoma, OV ovarian adenocarcinoma, CS chondrosarcoma, BRCA breast carcinoma, LGG low-grade glioma, ATRT atypical teratoid/rhabdoid tumor, GBM glioblastoma multiforme, THCA thyroid carcinoma, ACC adrenocortical carcinoma, TGCT testicular germ cell tumor, MESO mesothelioma, PFS progression free survival, OS overall survival, WT wildtype. Created with BioRender.com.

We investigated the prevalence of histone mutations across cancers and ages (Fig. 2), resulting in redefined histone mutation rates. Core histone mutations affected 10% of all subjects (9% core histone mutant only and ~1% co-occurring core and H1 mutant, Fig. 2a), and were most common among pediatric high-grade glioma/astrocytoma (pHGG, including diffuse midline glioma (DMG), *n* = 63, 61%), chondrosarcoma (*n* = 6, 67%), head and neck squamous cell carcinoma (HNSC, *n* = 103, 20%), and bladder carcinoma (BLCA, *n* = 77, 19%). Core histone mutations were also prevalent among B cell non-Hodgkin’s lymphoma (BNHL, *n* = 55 subjects, 24%). These BNHL cases included a subset harboring co-occurring core and H1 mutations (*n* = 20, 9% of entire BNHL cohort), indicating a larger-than-recognized role for histone alterations in lymphomas, beyond the established role for H1 mutations^20,21^. Across other cancers, H1 mutations were relatively rare, found in only 2% of subjects (Fig. 2a). H1 mutations occurred at the highest rates in BNHL (in addition to the previously mentioned 9% co-occurring core and linker histone mutants, another 7% of the BNHL cohort harbored linker histone mutations but no core histone mutations), esophageal carcinoma (ESO, *n* = 15, 5%), HNSC (*n* = 23, 4%) and BLCA (*n* = 18, 4%). With the exception of BNHL, <1.5% of subjects from any other cancer type harbored co-occurring core and linker histone mutations.

We also considered histone mutation rates in the context of patient age (pediatric, AYA, adult; Fig. 2b). Among the pediatric cancer population, histone mutations affected CNS tumors (primary CNS lymphoma, *n* = 1, 100%; HGG/astrocytoma, *n* = 55, 64%; neurofibroma/plexiform, *n* = 1, 17%; atypical teratoid/rhabdoid tumors (ATRT), *n* = 3, 12%; medulloblastoma, *n* = 6, 3%; ependymoma, *n* = 1, 3%; and low-grade glioma/astrocytoma (LGG), *n* = 5, 2%) and BNHL (*n* = 2, 12%). Among AYA subjects, in addition to CNS tumors (germinoma, *n* = 1, 100%; HGG, *n* = 9, 50%; GBM, *n* = 3, 10%; medulloblastoma, *n* = 3, 6%; LGG, *n* = 10, 3%) and BNHL (*n* = 6, 46%), histone mutations affected cancers including cervical squamous cell carcinoma and endocervical adenocarcinoma (CESC, *n* = 18, 22%), breast invasive carcinoma (BRCA, *n* = 15, 17%), HNSC (*n* = 3, 15%) and adrenocortical carcinoma (ACC, 3, 9%) (Fig. 2b). In adult cancers, histone mutation rates were generally higher when compared to pediatric and AYA, consistent with ageing-associated somatic mutagenesis^26,27^. Among adults, histone mutation rates were most elevated in BNHL (*n* = 64, 32%), HNSC (*n* = 123, 25%), BLCA (*n* = 94, 24%), ESO (*n* = 61, 22%), and CESC (*n* = 41, 20%). Histone mutations were less prevalent in adult CNS tumors (GBM, *n* = 23, 6%; LGG, *n* = 17, 6%) compared to pediatric entities.

### Clinical implications of core and linker histone mutations within cancer types

We investigated the clinical implications of histone mutations by performing Kaplan–Meier survival comparisons stratifying patients into histone mutant subtypes (e.g., core histone mutant, linker histone mutant, histone wildtype). Among patients diagnosed with ACC or uterine carcinosarcoma (UCS), the presence of a core histone mutation predicted a significantly shorter progression free survival (PFS) and overall survival (OS) when compared to histone wildtype patients (Fig. 2c, d). Among patients diagnosed with mesothelioma, H1 mutant cases exhibited significantly shorter PFS and OS outcomes (Fig. 2e). These data represent the first potential association of these cancer types with prognostic histone mutations. To understand whether histone mutations associated with previously defined prognostic subtypes, we compared histone mutation frequencies in molecular subtypes of ACC^28^, UCS^29^, and mesothelioma^30^ in the TCGA cohort. Among the three main ACC subtypes, the poor prognosis CoC3 subtype^28^ was enriched for core histone mutations (Chi-square, *p* = 0.0394, Supplementary Fig. 2, Supplementary Data File 2). Among UCS and mesothelioma, there were no significant enrichments of histone mutation frequencies among prognostic subtypes.

### Discovery of histone mutations in pediatric and AYA CNS tumors

**Fig. 3:**
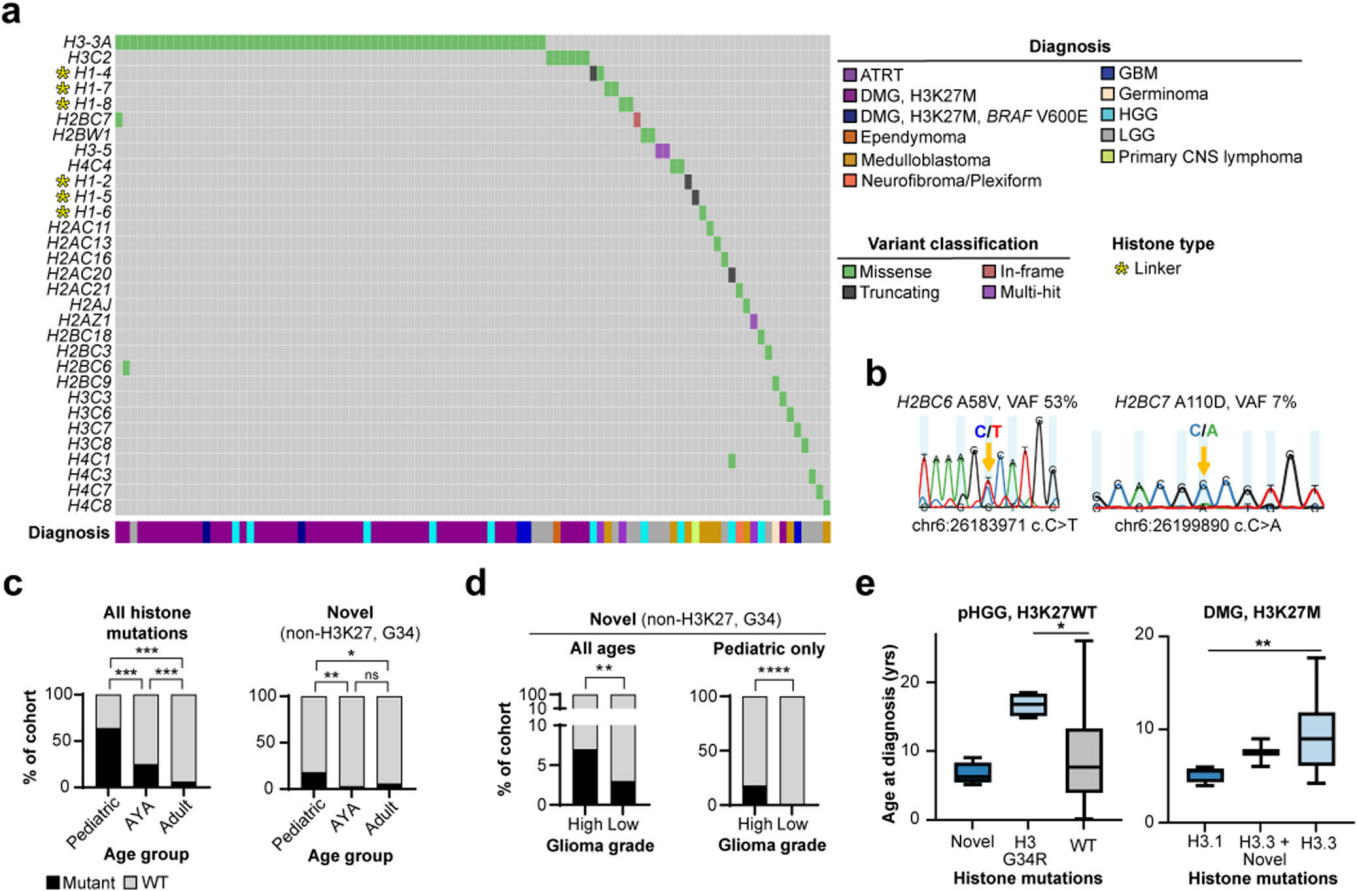
Expanded landscape of core and linker histone mutations in pediatric and adolescent CNS tumors. **a** Oncoplot showing histone gene alterations in children (0–14 years) and AYA (ages 14–39 years) diagnosed with CNS tumors. **b** Sanger sequencing validation of H2B mutations identified in H3.3 K27M mutant DMG patients (*top* = H2BC6 A58 mutation; *bottom* = H2BC7 A111 mutation). Gene names, amino acid changes, VAFs (detected by tumor whole genome sequencing), chromosome positions and nucleotide changes are listed for each mutation. Mutated bases are labeled and indicated by yellow arrows. **c** *Left:* Enrichment of all histone mutations (including H3 K27M and H3 G34R/V) in pediatric HGG and adolescent HGG/GBM, relative to adult GBM (Chi-square tests with Bonferroni correction). *Right:* Enrichment of additional (non-H3 K27 or H3 G34) histone mutations in pediatric HGG relative to AYA and adult HGG/GBM (Chi-square tests with Bonferroni correction; Fisher’s exact test used to compare AYA group given small [<5] sample size). **d** *Left:* Enrichment of histone mutation rate among high-grade (HGG, GBM) relative to low-grade (LGG) gliomas across the ages, excluding H3 K27M/H3 G34 mutant subjects (Chi-square test). *Right*: Comparison of histone mutation rates between pediatric HGG and LGG (Fisher’s exact test). **e** *Left:* Age at diagnosis of core histone mutant (H2A/H2B/H4 mutant, *n* = 4), H3 G34R mutant (*n* = 4) and histone wildtype (*n* = 40) pHGG. *P* < 0.05, Mann–Whitney test with Bonferroni correction. *Right:* Younger age at diagnosis of H3.1 K27M mutant (*n* = 5) relative to H3.3 K27M mutant (*n* = 47) DMG patients (*p* < 0.005, Mann–Whitney test with Bonferroni correction). Two subjects harboring co-occurring H2B/H3.3K27M mutations presented at a slightly younger age when compared to H3.3K27M-only DMG (n.s.). Abbreviations: VAF variant allele frequency, FS del frameshift deletion, DMG diffuse midline glioma, HGG high-grade glioma, LGG low-grade glioma, ATRT atypical teratoid/rhabdoid tumor, GBM glioblastoma multiforme.

Given the established oncogenic role for H3 tail alterations in pHGGs, the lack of previous investigations into non-H3 K27/G34 histone mutations in these cancers, and the relatively small number of somatic mutations in pediatric compared to adult solid tumors^26,27^, we closely examined the histone mutation landscape across pediatric and adolescent CNS tumors. We identified non-H3 K27/G34 core and linker histone mutations in cancers including ATRT, DMG, HGG, ependymoma and medulloblastoma (Fig. 3a). Notably, two cases of *H3-3A* K27M mutant DMG harbored core histone (H2B) mutations (a clonal and sub-clonal), both of which were subsequently validated by Sanger sequencing of the corresponding primary tumor-derived cell lines (Fig. 3b). In one of these cases, the mutated H2B-encoding gene (*H2BC6*) was among those histone genes harboring the highest average VAFs at pan-cancer level, consistent with clonality (Fig. 1g).

We did not identify additional histone gene alterations in H3 G34 mutant HGGs, however, the cohort size was limited (*n* = 8 cases). Among H3WT HGG, core histone mutations (H2A, H2B, and H4) were discovered in four subjects (hemispheric, *n* = 3; posterior fossa, *n* = 1). Intriguingly, a subset of rare pediatric brain tumors also harbored H1 mutations. This subset included the only pediatric primary CNS lymphoma case included in the study; one DMG, H3 K27-wildtype; medulloblastoma (*n* = 2); and ATRT (*n* = 2), representing the earliest report of H1 mutations in pediatric CNS tumors. Notably, the primary CNS lymphoma, DMG, and one medulloblastoma case each harbored H1 frameshift mutations, consistent with the high occurrence of this mutation type affecting the H1 family at pan-cancer level and in adult lymphomas (Fig. 1c). Our findings establish an expanded landscape of core and linker histone alterations in pHGGs and other pediatric CNS tumors, extending beyond the classical oncohistone mutations.

### Histone mutation rate in pediatric, AYA, and adult gliomas

Given the observed prevalence of histone mutations among pediatric and adolescent CNS tumors, in contrast to the relative rarity of these events in adult CNS tumors (Fig. 2b), we sought to define age and tumor type-specific enrichments for histone mutations. First, we compared histone mutation rates between pediatric, AYA, and adults diagnosed with HGG/GBM (histone mutation rate was defined as the percentage of histone mutant subjects divided by the total number of mutant and wildtype subjects). When considering all histone mutations, including H3 K27M and H3 G34R/V, there was a significant difference in histone mutation rate between each of these three age groups (*p* = 0.0003, Chi-square tests with Bonferroni multiple testing correction, Fig. 3c, *left*, Table 2). Given our interest in dissecting the prevalence non-H3 K27/G34 histone mutations across cancers and ages, we subsequently filtered out any subjects harboring only H3 K27M or H3 G34R/V histone mutations. Importantly, there remained a significantly higher histone mutation rate in pediatric relative to AYA (*p* = 0.0015, Chi-square test with Bonferroni correction), and in pediatric relative to adult HGG/GBM (*p* = 0.0417, Chi-square test with Bonferroni correction) when excluding these oncohistone H3 mutations (Fig. 3c, *right*, Table 2). We further compared histone mutation rates between high-grade (HGG, GBM) and low-grade gliomas (LGG) across all ages, again excluding H3 K27/G34 mutant subjects. Here, we observed a significantly higher rate of histone mutations in high- relative to low-grade gliomas (*p* = 0.0036, Chi-square test, Fig. 3d, *left*). When considering only pediatric subjects, this enrichment was even more pronounced (*p* < 0.0001, Fisher’s exact test, Fig. 3d, *right*).

**Table 2:** Comparison of histone mutation rates across ages and tumor grades among glioma patients.

All histone mutations (including H3 K27M and H3 G34R/V)						
	# mutant (%)	# WT (%)	Comparison	Test	*P*	Adjusted *P*^a^
Pediatric	55 (64%)	31 (36%)	Ped vs. AYA	Chi-square	<0.0001	0.0003, ***
AYA	12 (25%)	36 (75%)	Ped vs. Adult	Chi-square	<0.0001	0.0003, ***
Adult	23^a^ (6%)	335 (94%)	AYA vs. Adult	Chi-square	<0.0001	0.0003, ***
Histone mutations (excluding H3 K27/G34)						
	# mutant (%)	# WT (%)	Comparison	Test	*P*	Adjusted *P*
Pediatric	7 (18%)	31 (82%)	Ped vs. AYA	Chi-square	0.0005	0.0015, **
AYA	1 (3%)	36 (97%)	Ped vs. Adult	Fisher’s exact	0.0139	0.0417, *
Adult	22 (6%)	335 (94%)	AYA vs. Adult	Chi-square	0.4977	1

Top: Comparisons when considering all histone mutations, including H3 K27M and H3 G34R/V mutations. Bottom: Comparisons when excluding H3 K27M and H3 G34/RV mutations.

^a^Adj *P* = adjusted using Bonferroni multiple testing correction (multiply the *p*-value by number of statistical tests performed, *n* = 3). Fisher’s exact test used for cohort sizes <5.

Given the known associations between H3 K27M/G34 mutations and patient age at diagnosis, we next considered patient age in the context of histone mutation subtypes (e.g., non-H3 K27/G34 histone mutant pHGG/DMG). In alignment with published data^4^, H3 G34 mutations affected significantly older children/adolescents (median 16.8 years, *n* = 4) when compared to histone wildtype HGG (median 7.6 years, *n* = 40; *p* = 0.043, Mann–Whitney test with Bonferroni multiple testing correction, Fig. 3e, *left*). HGGs harboring core histone mutations beyond H3 K27/G34 (H2A, H2B, H4; *n* = 4) presented at a younger age (median 6.3 years, *n* = 4) than H3 G34 mutants (n.s., *p* = 0.086, Mann–Whitney test with Bonferroni multiple testing correction). Among patients diagnosed as H3 K27M mutant DMG, those harboring H3.1K27M mutation (median 5.5 years, *n* = 5) presented at a younger age than H3.3 K27M mutants (median 9.0 years, *n* = 47; *p* = 0.0051, Mann–Whitney test with Bonferroni multiple testing correction, Fig. 3e, *right*), again consistent with previous findings^4^. The two DMG cases harboring co-occurring H3.3 K27M and H2B mutations presented at a median age of 7.5 years. These two patients also trended towards a slightly shorter overall survival (OS) outcome relative to H3 K27M mutant DMGs that did not harbor additional histone mutations (median OS 7.2 months *vs*. 11.9 months, respectively; *p* = 0.079, log-rank Mantel–Cox test, Supplementary Fig. 3). While these double histone mutant DMG cases were rare, our findings warrant expansion to a larger cohort of pHGG/DMG patients to define whether those harboring additional core histone mutations may represent a distinct molecular/clinical subtype.

### Mutual exclusivity and co-occurrence of histone gene mutations

**Fig. 4:**
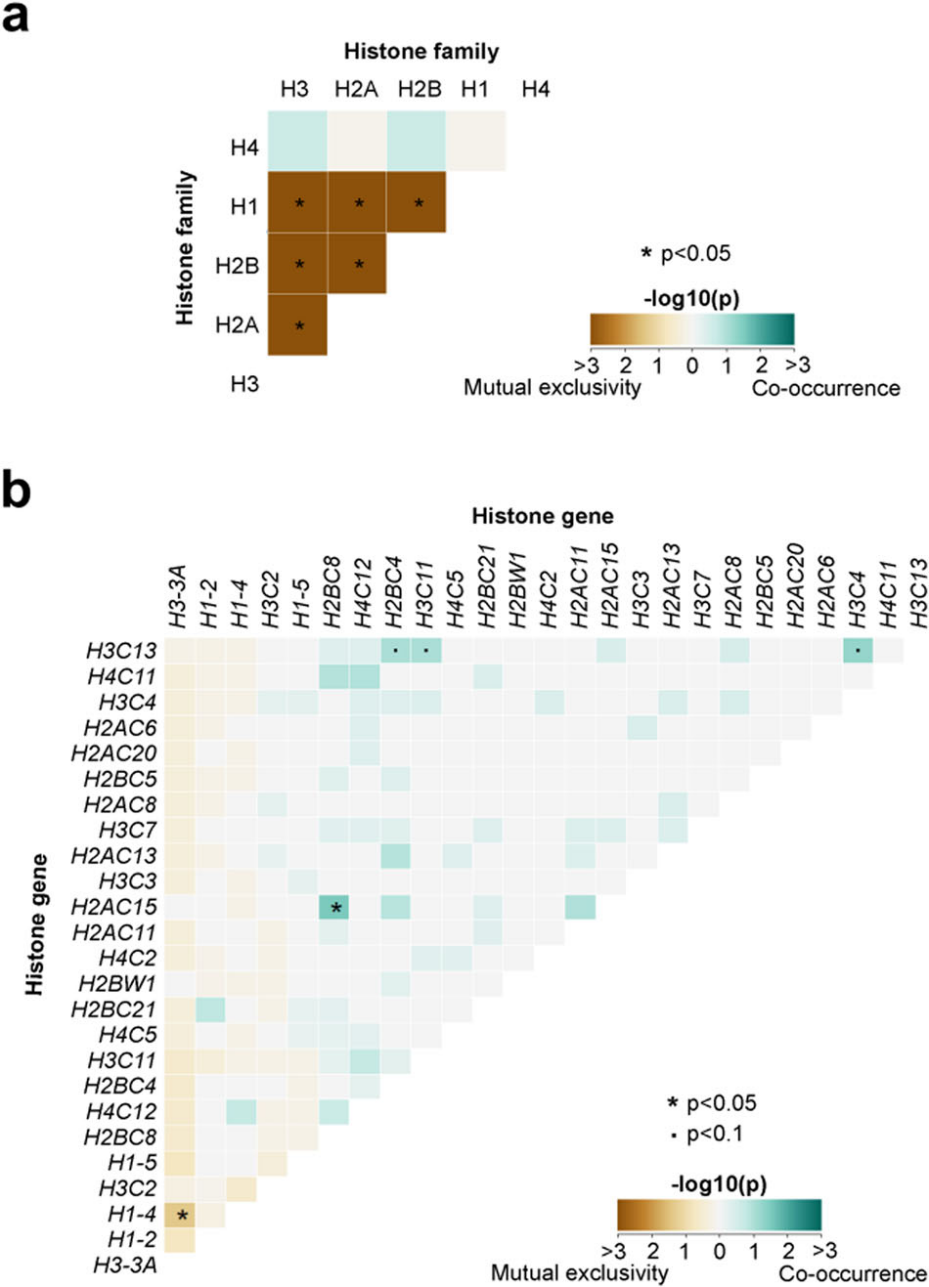
Mutual exclusivity and co-occurrence of histone family and histone gene mutations. Results of Fisher’s Exact test for mutual exclusivity or co-occurrence of mutations in histone gene families (**a**) and in pairs of histone genes (**b**). Significant, *p* < 0.05.

We next assessed mutual exclusivity and co-occurrence of histone mutations using Fisher’s exact tests to identify histone genes, and histone gene families, that were co-mutated more or less frequently than expected by chance. Grouping together all histone genes belonging to a single histone family (i.e., all H1-encoding genes were classified as a single group, ‘H1’) revealed that mutations affecting each histone family (H1, H2A, H2B, H3) tended towards mutual exclusivity (Fig. 4a). H1 mutations were mutually exclusive with H2A, H2B, and H3 mutations; H2A and H2B mutations were mutually exclusive with one another; and both H2A and H2B were mutually exclusive with H3 mutations. When considering individual genes, the analysis was restricted to the top 25 most mutated histone genes. Intriguingly, *H3-3A* and *H1-4* mutations were mutually exclusive across cancers, whereas *H2BC8* and *H2AC15* significantly co-occurred (Fig. 4b).

### Histone gene mutation bias and proliferation-associated codon usage

Next, we investigated why certain histone genes (e.g., *H3-3A*) were highly mutated across cancers while other genes harboring the same or similar protein sequences were rarely mutated. This mutational bias might be explained by differential codon usage across histone genes. Genes that are highly expressed in proliferating, but not in differentiated, cells exhibit a proliferation-associated codon signature (i.e., their mRNA sequence consists of codons optimal for rapid translation in proliferating cells, with corresponding tRNAs abundant in the cytosol of dividing cells; accordingly, these genes can be rapidly and accurately translated into proteins^31^). Previous work has shown that recurrently mutated cancer driver genes harbor proliferation-associated codon signatures^31,32^. A comparison between *H3-3A* and *H3-3B*, which encode identical protein sequences with different 5’ untranslated regions^33^, revealed that *H3-3A* harbors a more proliferation-associated codon signature when compared to *H3-3B*^34^, in keeping with their different somatic mutation burdens as confirmed by our study (Fig. 1a).

**Fig. 5:**
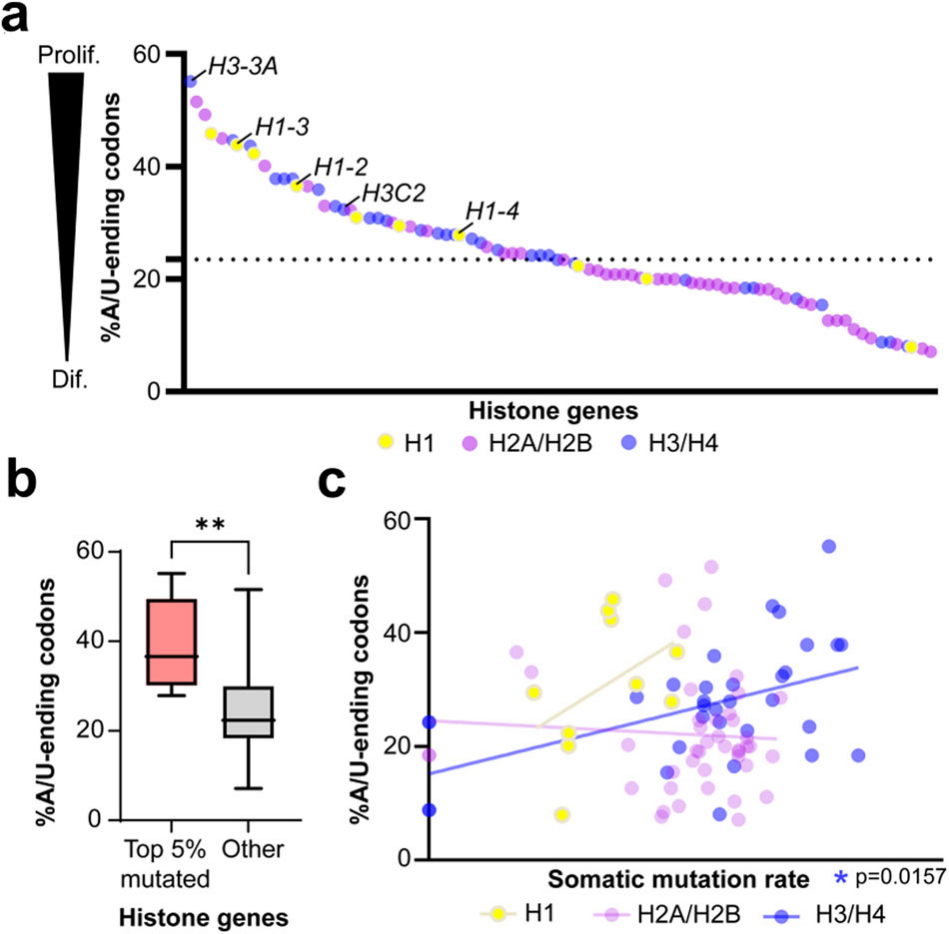
Histone gene codon usage associates with somatic mutation rate. **a** The percentage of A/T-ending codons (%A/T-ending, y-axis) in the coding sequence of each histone gene (*n* = 88). Genes with higher %A/T-ending codons=proliferation-associated (‘Prolif’); genes with lower %=differentiation-associated (‘Dif’). Histone genes are divided by family (H1, H2A/H2B, and H3/H4), and the top five histone genes with the highest mutation burdens are labeled. The dotted line (y-axis) represents the median %A/T-ending codons across all histone genes (23.5%). **b** Higher %A/T-ending codons in the top five most mutated histone genes (labeled in **a**) relative to all other histone genes, *p* = 0.0047, Mann–Whitney test. Box plots show the upper and lower quartiles and the median. **c** Correlation between %A/T-ending codons (y-axis) and somatic mutation rate (log_10_(*n* + 1)/CDS, x-axis), in the H1, H2A/H2B, and H3/H4 histone families. H3/H4, *p* = 0.0157, Spearman correlations.

We built upon these existing data by investigating mRNA codon usage across all histone genes. We computed the percentage of codons ending in A or T(U) (‘%A/T-ending’), a signature associated with highly expressed genes in proliferating cells^31,32^. *H3-3A* emerged as having the most proliferation-associated codon signature of all histone genes (i.e., highest %A/T-ending codons, 2.3-fold compared to median across all histone genes [55% for *H3-3A* relative to median of 23.5%], Fig. 5a). Other highly mutated histone genes (e.g., *H3C2* and several H1-encoding genes) similarly exhibited a proliferation-associated codon bias. In fact, the %A/T-ending codons of the top 5% most mutated histone genes (*H3-3A, H3C2, H1-2, H1-3, H1-4*) were significantly higher when compared to other histone genes (*p* = 0.0047, Mann–Whitney test, Fig. 5b). Moreover, we correlated %A/T-ending codons to somatic mutation rates (log_10_(*n* + 1)/CDS, with *n* = mutation count and CDS = gene coding sequence length). Among H3/H4 genes, there was a significant correlation between %A/T-ending codons and somatic mutation rates (*p* = 0.0157, Spearman’s correlation, Fig. 5c). In contrast, only a weak trend existed between %A/T-ending codons and mutation rate among H1 genes (perhaps due to the smaller number of H1 genes included for analysis, *n* = 10; *p* = 0.166), and there was no correlation among H2A/H2B genes. Our findings point to distinctions in mutation selection between histone families and suggest that cancer cells preferentially mutate histone H3/H4 genes that are efficiently synthesized in proliferating cells, thus potentially resulting in rapid incorporation of oncohistones into chromatin.

### Pan-cancer recurrent mutation hotspots affected evolutionarily conserved and functional histone residues

**Fig. 6:**
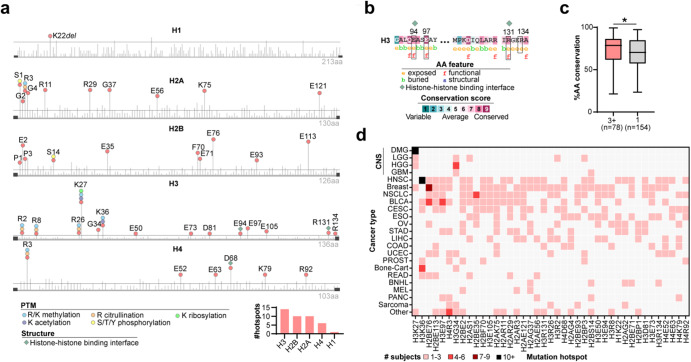
Pan-cancer recurrent mutation hotspots affect functional and highly conserved positions of the histone protein. **a** Lollipop diagrams show the position along the protein sequence of mutations affecting genes of each histone family (H1, H2A, H2B, H3, H4). Hotspot mutations (mutated in ≥6 cancer genomes) are labeled. Horizontal line indicates threshold of 5 mutation events. PTM at each residue is indicated by colored circles. Histone-histone binding interfaces are marked with green diamonds. Protein length is indicated (e.g., 213AA). *Bottom right:* bar graph shows the number of unique hotspots affecting each histone family. **b** Examples of MSA showing mutation hotspots that affect the most conserved positions of the histone protein, including functional residues (high sequence conservation and position exposed at protein surface). **c** Comparison of the amino acid (AA) conservation frequency (MSA) across species (‘%AA conservation’, y-axis), of histone residues mutated in 3+ genomes (*n* = 80) versus those mutated in only 1 genome (*n* = 191; *p* = 0.0155, Mann–Whitney test). Box plots show upper and lower quartiles and the median. **d** Distribution of hotspot mutations across cancers. Colors indicate the number of subjects belonging to each cancer type that harbor a mutation at the indicated mutation hotspot. Abbreviations: PTM post-translational modification, AA amino acid, VAF variant allele frequency, MSA multiple sequence alignment.

We identified the 5% most recurrently mutated histone residues (i.e., altered in ≥6 samples), and designated these residues as “histone mutation hotspots”. Our analyses revealed 41 recurrent hotspots (Fig. 6a). The greatest number of unique hotspots affected H3 histone genes (*n* = 14 hotspot mutations), whereas only one hotspot was present among H1 histone genes (K22del). Mutation hotspots included both known oncohistones (H3 K27M, H3 K36M), and yet-uncharacterized N terminal tail-altering mutations across all four core histone families, several of which altered epigenetically modified sites (Fig. 6a). Other mutation hotspots (H3 E94, H3 R131, H4 D68) affected key functional residues including histone-histone binding interfaces, suggesting that these mutations disrupt nucleosome binding and structural integrity, consistent with recently reported oncohistones^12,13^.

We evaluated whether mutation hotspots affected more evolutionarily variable, or conserved, positions of the histone protein using multiple sequence alignment (MSA). Several hotspots affected the most evolutionarily conserved positions of the histone protein, including functional residues (examples are shown in Fig. 6b). There was a significant association between histone residue mutation frequency and evolutionary conservation (percentage conservation across species): recurrently mutated residues (mutated in three or more cancer genomes) were more conserved across species when compared to residues that were mutated in only one genome (*p* = 0.0155, Mann–Whitney test, Fig. 6c). This finding provides support that recurrent mutations targeted highly conserved, functionally or structurally important residues, rather than randomly affecting variable positions of the histone protein.

We next assessed the occurrence of mutation hotspots across cancers, to identify whether these events were specific to a given tumor type or occurred more broadly across cancers. Hotspots exhibited known cancer type specificity such as H3 K27M (DMG), H3 G34R/V (pHGG), H3 K36M (HNSC, bone and cartilage tumors), and associations including H2B E76 (breast cancer, bladder cancer), H2B E35 (non-small cell lung cancer), and H3 E97 (bladder cancer) (Fig. 6d). However, many hotspots were distributed broadly across cancers, and even those associated with a given cancer type (e.g., H3 G34 to HGGs) were found to occur at a lower frequency in several other cancers. For example, H3 G34 mutations were found in ACC and uterine cancers, and H3 K27 mutations were found in breast (H3 K27R) and AML (H3 K27M) subjects (Fig. 6d). Thus, while hotspot histone mutations may be prominent features of a certain cancer type, they can also be found in rare cases of other tumor types^35^, providing a common link between cancers.

### Core histone mutations largely resulted in loss of charged amino acids

**Fig. 7:**
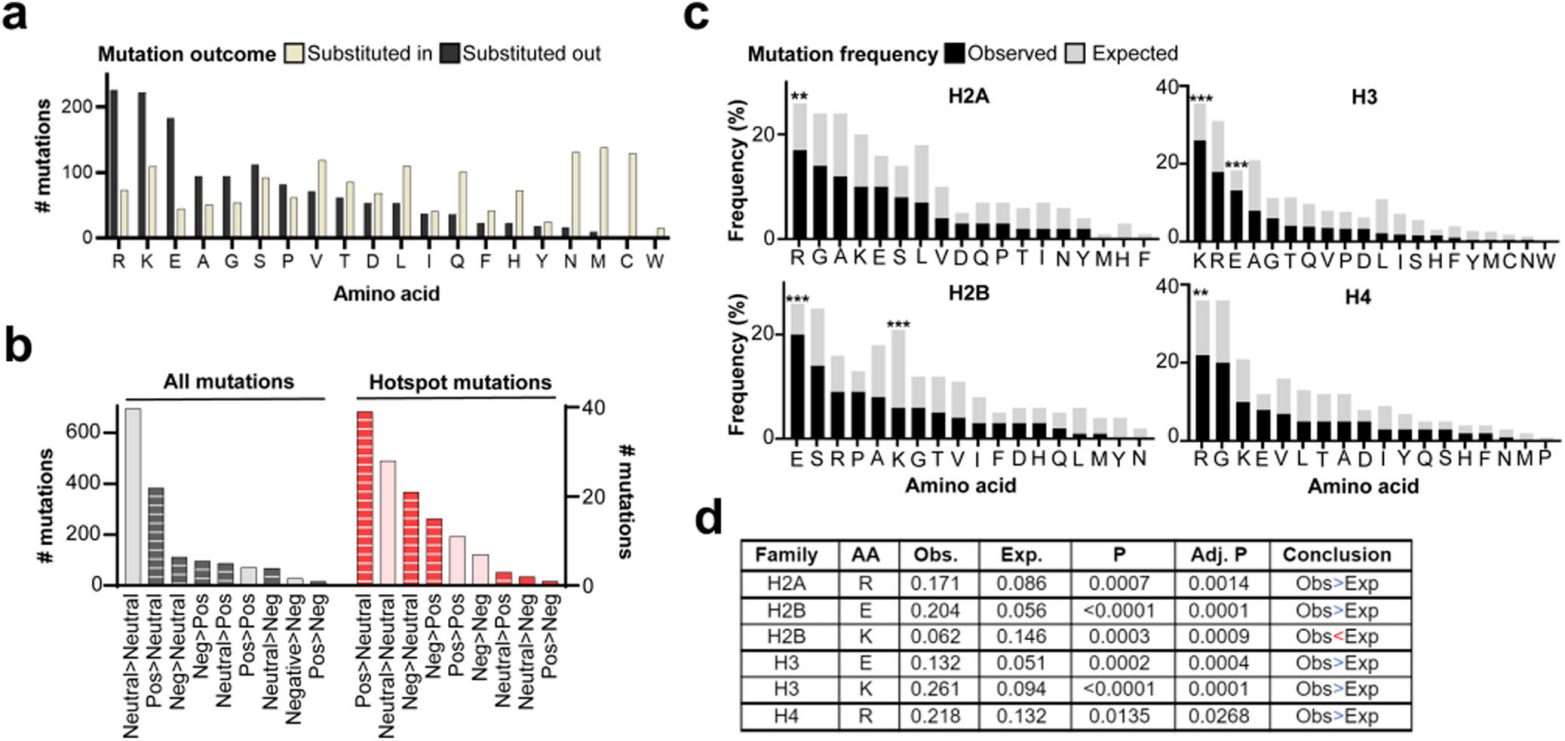
Enrichment of missense mutations resulting in loss of charged residues among core histone proteins. **a** Number of missense mutation events resulting in substitution out (black) and substitution in (tan) of each amino acid into the mutant histone protein. **b** Charge changes resulting from all missense mutation (*left*) and from hotspot mutations (*right*). Solid = conservative change, striped = nonconservative change. **c** Comparison between observed (black) and expected (gray) mutational frequencies affecting each amino acid among core histone families. **d** Table summarizing the significant results from **a**, showing a difference in the observed versus expected mutation frequencies affecting amino acids in each core histone family. Adjusted (adj.) *p*-values were adjusted to account for the number of amino acids tested in the analysis. Amino acids for analysis were chosen based on visual observation of different expected versus observed frequencies. Conclusions are based on adj. *p*-values. Conclusion ‘Obs>Exp’ = observed frequency of mutations is significantly greater than the expected frequency; Obs<Exp = observed is significantly lower than expected frequency. Abbreviations: AA amino acid, pos positively charged, neg negatively charged amino acid.

Subsequently, we investigated amino acid changes resulting from histone mutations to define patterns of biochemical changes affecting mutant histones. Missense mutations largely resulted in loss of charged amino acids (lysine, arginine, glutamic acid) and introduction of neutrally charged residues (e.g., asparagine, cysteine, methionine) into the mutant histone protein (Fig. 7a). Indeed, the most frequently occurring amino acid changes were non-conservative substitutions (Supplementary Fig. 4A). This pattern was especially pronounced among hotspot histone mutations, which included a higher percentage of non-conservative mutations when compared to all missense mutations (i.e., including non-hotspots; Supplementary Fig. 4B, Fig. 7b). To define this trend more closely and within individual core histone families (H2A, H2B, H3, H4), we compared the ‘expected’ versus ‘observed’ mutation rate affecting each amino acid, within each histone family. We calculated the frequency at which each amino acid occurred in histone protein coding sequences among each histone family and considered this value to be the ‘expected’ mutational frequency if all amino acids were mutated equally and randomly. We compared the expected to the observed frequency (the actual number of missense mutations affecting each amino acid, divided by the total number of missense mutations affecting all amino acids). Kolmogorov-Smirnov tests for equality of distributions were used to compare the expected to the observed mutational frequencies.

This analysis revealed a significantly higher than expected frequency of missense mutations altering glutamic acid (E) residues in H3 histones, arginine (R) mutations in H2A and H4 histones, and lysine (K) mutations in H2B and H3 histones (Fig. 7c, d). The statistical over-representation of these mutations suggested selection for mutations altering E, R, and K histone residues in cancer genomes. This finding, together with the observation that missense mutations largely substituted in neutrally charged amino acids (Fig. 7a), pointed to a putative mechanism by which missense mutations may disrupt electrostatic interactions between histone proteins and DNA, thereby altering nucleosome structure, stability, and turnover dynamics^15^.

### Validation of pan-cancer histone mutation patterns in an independent cohort of cancer cell lines

**Fig. 8:**
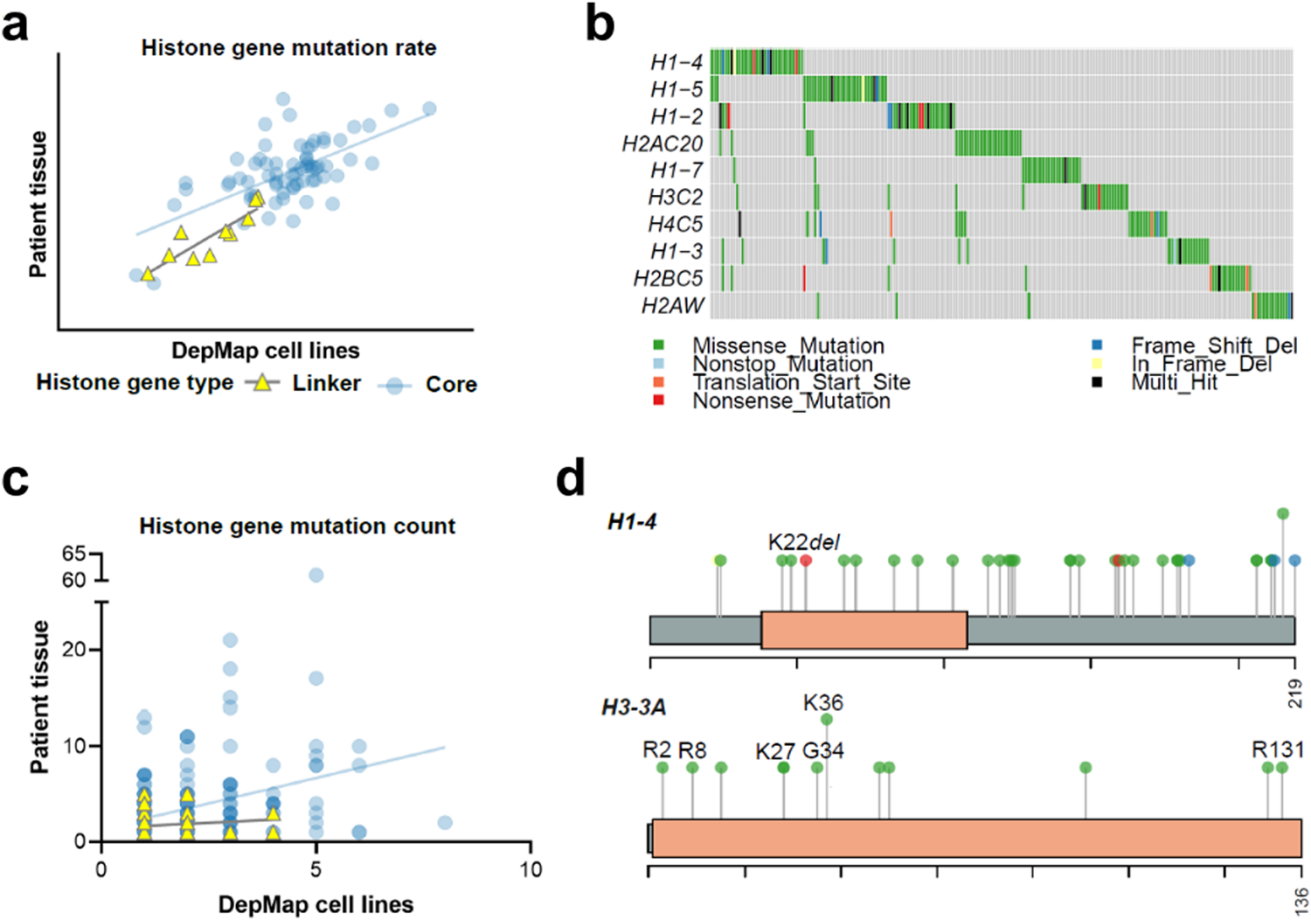
Overlapping histone mutation spectrum between patient tumor tissue and DepMap cancer cell lines. **a** Correlation between histone gene mutation rate in DepMap cancer cell lines (x-axis) and patient tissue (y-axis). Mutation rate = log_10_(*n* + 1)/CDS; *n* *=* mutation count, CDS coding sequence length. **b** Oncoplot showing the top ten most recurrently mutated histone genes among the DepMap cell lines. **c** Correlation between the missense mutation count affecting each histone residue (e.g., H3 K27) between DepMap cancer cell lines (x-axis) and patient tissue (y-axis). Each circle represents an individual histone residue, and the axes show the missense mutation count at that residue for each dataset. **d** Lollipop diagrams show mutations affecting the top-mutated gene *H1-4*, including the hotspot K22del, as well as *H3-3A* (bottom), highlighting the presence of several patient tissue-defined hotspot mutations (H3 R2, R8, K27, G34, K36, R131) among DepMap cancer cell lines. Amino acid length is labeled for each protein (219 and 136 amino acids for *H1-4* and *H3-3A*, respectively). Color-coding of lollipop circles is consistent with **c**.

To more closely investigate the functional effects of core and linker histone mutations in cancer, we leveraged functional genomics data from The Cancer Dependency Map (DepMap) project, consisting of whole exome sequencing (WES) and drug sensitivity data. First, we validated the histone mutational patterns defined by patient tissue (from the previously described tumor tissue cohort, *n* = 12,743) in the independent cohort of DepMap cancer cell lines (*n* = 1703) to determine the extent of overlap in the histone mutational spectra between these primary tumors and in vitro cancer model systems. Exome sequencing data was used to identify nonsynonymous core and linker histone mutations among cancer cell lines. Mutation data were filtered to include only rare variants absent from the population or with <0.01 maximum population frequency according to the Genome Aggregation Database (gnomAD). The mutation rates affecting each histone gene (log_10_(*n* + 1)/CDS) in patient tissue and in DepMap cell lines were assessed for correlation. Histone gene mutation rates strongly correlated between the two datasets (*p* = 0.0008 and *p* < 0.0001 for linker and core histone genes, respectively; simple linear regression; Fig. 8a). In addition, we queried the top-most mutated histone genes among DepMap cell lines and found that H1 genes *H1-2/4/5* were the three most recurrently mutated histone genes (Fig. 8b). The H3-encoding gene *H3C2*, but not *H3-3A*, was among the top ten most mutated histone genes. The absence of *H3-3A* is likely due to the under-representation of pediatric CNS tumor cell lines in the current DepMap resource, in contrast to our patient tissue cohort which included many pHGG/DMGs with H3 G34R/V and H3 K27M mutations, respectively.

Next, we studied individual histone residues that were mutated and assessed concordance of missense mutation counts affecting each residue between the two datasets. We considered core and linker histone missense mutations separately. In keeping with our observation that linker histone mutations occurred sporadically across the H1 protein, there was no appreciable relationship between missense mutation rate affecting each H1 residue between patient tissue and DepMap cell lines (*p* = 0.31, simple linear regression, Fig. 8c). In contrast, the rate of missense mutations affecting core histone residues significantly correlated between patient tissue and DepMap cell lines (*p* < 0.0001, Fig. 8c), revealing that the histone mutational landscapes were consistent between these two datasets. Moreover, the mutational patterns affecting different histone families was consistent with the results among patient tissue, including missense mutations and frameshift/disruptive mutations broadly distributed across the globular and C-terminal domains of the H1 protein (Fig. 8d). We also identified 34 out of 41 (83%) of the mutation hotspots defined by patient tissue to be present in cancer cell lines. These hotspots included, for example, mutations in H3 genes (R2, R8, K27, K36, G34 and R131) (Fig. 8d).

One notable difference between the two datasets was that the overall histone mutation rate was higher among DepMap cell lines when compared to patient tissue (e.g., 10% of DepMap cell lines were linker histone mutants, compared to only 2% of patient tissue). This difference may be attributed to more stringent upstream data processing among the patient tissue cohort (see Methods); the inclusion of more pediatric cases in the tumor tissue cohort relative to the DepMap cell lines, as the latter consisted primarily of adult cancer cell lines; and/or an increased acquisition of mutations when cells were propagated in culture, which may include accumulation of passenger mutations. Despite this difference, our collective results revealed that cancer cell lines provided a faithful model system through which to interrogate the functional effects of core and linker histone mutations in cancer.

#### Histone mutant cancer cell lines exhibit distinct therapeutic vulnerabilities

**Fig. 9:**
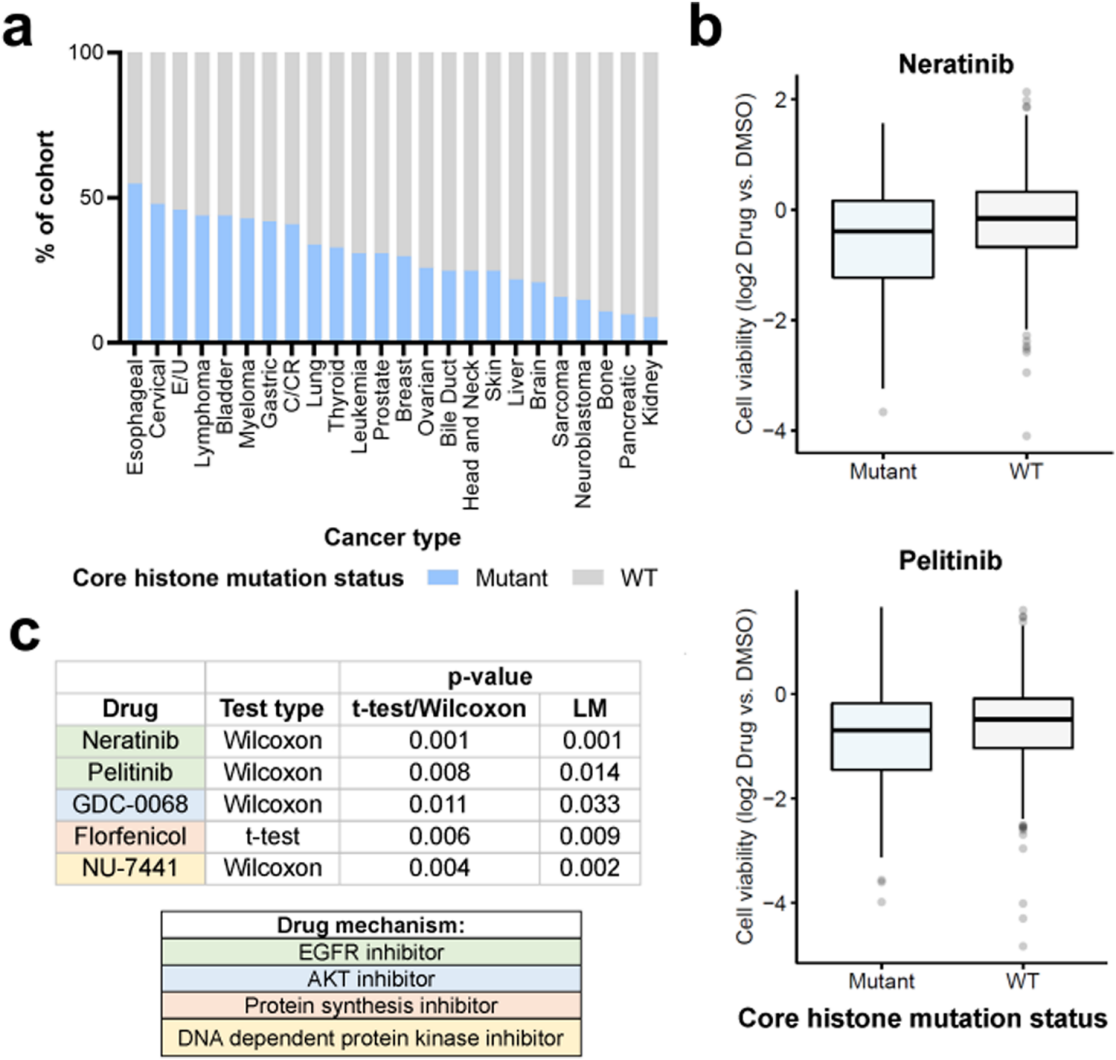
Core histone mutation status associates with drug response profiles. **a** Cancer type of cell lines included for the analysis, filtered to include only cancer types with ≥5% core histone mutants (and with number of histone mutant cell lines, *n* > 3). **b** Summary table listing examples of drugs for which core histone mutant cancer cell lines exhibited significantly higher sensitivity when compared to core histone wildtype cell lines. Significance was determined using t-tests with Welch’s correction (parametric data) or Wilcoxon rank sum tests (non-parametric data); and using linear models (LM) incorporating core histone mutation status, cancer type, sex, and age of the donor patient. **c** Box plots comparing drug response (viability of cells treated by drug relative to DMSO control, y-axis) between core histone mutant (blue) and WT (gray) cancer cell lines. Box plots show upper and lower quartiles and the median. Abbreviations: WT wildtype, E/U endometrial/uterine cancer, C/CR colon/colorectal cancer, EGFR epithelial growth factor receptor, AKT protein kinase B, LM linear model.

Having established that cancer cell lines provided a model system representative of patient tumor tissue, we sought to delineate the effects of histone mutations on cancer cell drug response profiles using the PRISM drug repurposing resource. We focused on core histone mutants given the greater prevalence of core relative to linker histone mutations in cancer (Fig. 1). We excluded cell lines from cancer types that harbored few or no histone mutations, to focus on cancer types that harbored a higher percentage of core histone mutant cell lines (≥5% core histone mutants, Fig. 9a). We performed a preliminary discovery analysis (see Methods) to identify candidate drugs with different response profiles between core histone mutant and wildtype cells. Drugs of interest, based on lowest p-values in the discovery screen, were then selected for manual comparison between mutant and wildtype cells using t-tests with Welch’s correction or Wilcoxon rank sum tests (see Methods for details). Subsequently, drugs of interest with significantly different response profiles using t-tests/Wilcoxon tests were selected for analyses in linear models incorporating histone mutation status, donor patient cancer type, age and sex. Only drugs that remained significant predictors of drug response (*p* < 0.05) when incorporating these clinical covariates into the linear model were considered. We focused on drugs that were more effective at targeting core histone mutant cancer cells and found that these cells exhibited significantly higher sensitivities to several biologically relevant repurposed and/or cancer drugs, including multiple epithelial growth factor receptor (EGFR) inhibitors (e.g., neratinib, pelitinib, Fig. 9b, c) and other drugs targeting cell growth pathways, indicating a potential therapeutic strategy.

## Discussion

Given the growing body of literature pointing to an expanded role for cancer-associated histone mutations, we established a detailed atlas of somatic mutations altering core and linker histone proteins across ages and cancers. Although we investigated histone mutations in >12,700 pan-cancer genomes, one limitation of our study was that our cohort overrepresented adult relative to pediatric subjects, mainly due to inherently larger numbers of adults than children diagnosed with cancer. We mitigated this issue among CNS tumors by amassing >800 pediatric and adolescent CNS tumor genomes from the PBTA. Our analyses revealed a significant enrichment of histone gene mutation rates among pediatric/AYA HGG relative to adult GBM, and in pediatric/AYA HGG relative to LGG. These findings highlight a special role for histone alterations in the context of high-grade CNS tumors in children and justify expansion to other cancers to define whether associations between histone mutation rate and patient age are tissue-specific, or perhaps override tumor type and represent a shared feature of pediatric cancers.

Analyses of the codon signature of histone genes revealed a significant correlation between the percentage of proliferation-associated (A/T-ending) codons and somatic mutation rate among H3/H4 histone genes. We interpreted this association to signify that H3/H4 genes that were more actively synthesized/incorporated into chromatin in rapidly growing cancer cells, were in turn preferentially selected for somatic mutation as the effects of mutation would be more profound due to rapid and dynamic integration into chromatin. This correlation existed in H3/H4, but not H2A/H2B, histone genes; one explanation for this difference is that H3/H4 tetramers are less stable, and inherently more dynamically incorporated into the nucleosome^36^. Thus, just as H3/H4 exhibit distinct nucleosome dynamics, they may also be affected by distinct translational dynamics in cancer cells which drive selection of pro-proliferative histone genes.

We were intrigued to find an absence of recurrent mutation hotspots affecting H1-encoding genes, with the exception of one deletion event (H1 K22del). Rather, mutations affecting H1 proteins were distributed broadly across the H1 coding region with a prevalence of disruptive frameshifts and deletion events. The H1 mutational pattern we uncovered at pan-cancer level aligns with previous studies of H1 mutations in adult lymphoma, wherein these mutations are driver events resulting in chromatin decompaction and oncogenic gene expression^17,20,21^. Importantly, we showed that H1 mutations are not restricted to lymphomas but are a feature of several previously unassociated pediatric and adult cancers. Our findings highlight a growing role for pan-cancer H1 mutations, warranting future investigations into the biological role of linker histone disruption across ages and malignancies.

While we focused on mapping the genomic landscape of somatic protein-altering histone mutations across cancers, it would be worthwhile to investigate the potential role for germline histone mutations and additional somatic histone gene alterations, including copy number variations, splice variants and non-coding variants, which may affect histone protein production and/or incorporation into the nucleosome. For example, a subset of pHGGs harbor gains of chromosome arm 1q^4^ which encodes several histone-encoding genes including *H3-3A*. Interrogation of histone gene copy number variations, particularly when they coincide with mutant histone alleles (e.g., *H3-3* K27M), may provide insight into the interplay between histone gene mutation and genome structure (including dynamics of mutant histone incorporation into chromatin and effects on tumor epigenetic patterning), resulting in improved understanding of histone and chromatin alterations in cancer.

The implications of histone mutation status on drug sensitivity profiles among the DepMap cancer cell lines points to a potential therapeutic opportunity for targeting histone mutant cancers. In particular, our findings suggest that histone mutant cancer cells are more sensitive to drugs targeting certain cell growth signaling pathways, including the EGFR pathway. One potential explanation for the observed heightened sensitivity of histone mutant cancer cells to these drugs could be a co-occurrence of histone and partner gene mutations affecting key growth factor signaling genes. Perturbed chromatin states resulting from histone mutations could also affect the accessibility of genes involved in growth factor signaling pathways, resulting in altered treatment responses. Our findings warrant further investigation to delineate the effects of histone mutations on cancer cell drug response profiles. Uncovering potential treatment strategies for histone mutant tumors would be particularly relevant for cancers enriched in histone mutations (e.g., pHGG) and for cancers where we identified associations between histone mutation status and patient survival outcomes (e.g., adrenocortical carcinomas).

## Conclusions

Our large-scale analysis of pan-cancer genomes generated a redefined landscape of somatic core and linker histone mutations across cancers and ages. These findings included discovery of core and linker histone mutations in rare pediatric brain tumors, and identification of the clinical relevance of these mutations in adult malignancies that are typically not screened for histone gene alterations.

## Methods

### Patient cohort for analysis

Whole genome and/or whole exome sequencing data from 12,743 patient-matched tumor and germline genomes were obtained from The Cancer Genome Atlas (TCGA, https://www.cancer.gov/tcga) version MC3 (*n* = 10,131; WGS: 60× tumor, 30× normal; WES: 200×), the International Cancer Genome Consortium Pan-Cancer Analysis of Whole Genomes (ICGC PCAWG) consensus SNV/indel release (*n* = 1798; WGS: mean 38× and 60× tumor, bimodal; 39× normal)^23^, and the Pediatric Brain Tumor Atlas (PBTA) version r18 (*n* = 814; WGS: 60× tumor, 30× normal)^24^. Where available, patient clinical and demographic data were obtained from the same sources.

### Upstream processing of tumor mutation data

TCGA, ICGC PCAWG, and PBTA datasets were analyzed to identify somatic alterations present in tumor tissue and absent from patient-matched germline tissue. A consensus variant calling approach was applied such that only somatic variants called by two or more pipelines were retained for analysis. For all variant call data, a threshold of 5% variant allele frequency (VAF) was set to filter out low frequency mutation calls and putative technical artifacts. Histone gene mutations in hypermutant tumors were excluded. Nonsynonymous mutations were queried across 88 histone-encoding genes based on the Hugo Gene Nomenclature Committee (HGNC) Group ID 864 (histone gene list as per January 2021). Genes flagged as pseudogenes at the time of gene list download were excluded from the analysis.

### Visualization of mutation data

The R package maftools^37^ was used to generate mutation oncoplots, lollipop plots, and VAF plots.

### Sanger sequencing

Genomic DNA was isolated from 5 × 10^5^ viable cells (cell line IDs CNMC-D-1034 and 7316-195) using Qiagen AllPrep DNA/RNA mini kit. DNA was quantified using Nanodrop or Qubit Broad Range Assay kit. DNA (200 ng) was sent to an outside vendor (GENEWIZ, LLC) for Sanger sequencing of the target region. Chromatogram files were analyzed using Indigo SNV and InDel Discovery (https://www.gear-genomics.com)^38–40^.

### Mutual exclusivity and co-occurrence of mutations

The R package maftools^37^ was used to generate mutual exclusivity and co-occurrence plots, using the somaticInteractions function to perform pair-wise Fisher’s exact tests to detect significant mutually exclusive or co-occurring gene pairs among the top 25 most mutated histone genes. Significance was assigned at a threshold of *P* < 0.05.

### Histone gene codon composition and somatic mutation rate

Histone gene coding sequences (mRNA) were downloaded from ENSEMBL^41^. The number of occurrences of each codon was computed. The number of codons ending in A or T(U), ‘A/T-ending’, was divided by the total number of codons in the mRNA sequence, to obtain the ‘%A/T-ending codons’. %A/T-ending codons was compared to the somatic mutation rate for each histone gene. Mutation rates were calculated as: log_10_(*n* + 1)/CDS, where *n* *=* mutation count, CDS=coding sequence length, and a pseudo-count of +1 was used to account for genes with zero mutations. CDS lengths were downloaded from ENSEMBL. Log scale was used to normalize for the high level of variance in genes with low mutation counts. A pseudocount of +1 was used to account for genes with no mutations. Spearman correlation was used to correlate %A/T-ending codons with somatic mutation rate.

### Calculating observed versus expected amino acid mutation rate

The expected mutational frequency affecting each amino acid was determined by calculating the frequency at which that amino acid occurred in all proteins comprising the H2A, H2B, H3, and H4 histone families separately. FASTA protein sequences were obtained from UniProtKB. In cases where *n* different histone genes encoded the same protein sequence, that sequence was represented *n* times when calculating the amino acid frequency. For example, *H3-3A* and *H3-3B* genes encode the same protein sequence; when calculating the amino acid frequency in H3 proteins, the sequence was counted twice. The observed frequency was calculated as the number of mutations affecting a given amino acid, divided by the total number of observed mutations affecting the H2A, H2B, H3, and H4 histone families separately. The distributions of expected versus observed mutation frequencies for amino acids of interest were compared using Kolmogorov-Smirnov tests for equality of distributions. Amino acids to test were chosen based on visual observation of different observed and expected mutation frequencies. *P*-values were adjusted based on the number of amino acids tested for. Statistical analyses were reviewed by a biostatistician (H.G.D.).

### Annotating histone protein features

Histone DNA interfaces were identified from published data^42^. Post-translational modifications (PTM) were derived from HISTome2: The Histone Infobase^43^. Lollipop mutation plots were created using the R package G3viz^44^.

### Multiple sequence alignment

Multiple sequence alignments (MSA) were performed using the Consurf Server (https://consurf.tau.ac.il/)^45–47^ with protein FASTA sequences obtained from UniprotKB^48^. MSA were constructed using the HMMER homolog algorithm with default parameters: e-value cut off 0.0001; UNIREF-90 protein database; automatic selection of homologs; 150 sequences that sample the list of homologs; maximum 95% and minimum 35% identify between sequences; MAFFT-L-INS-I alignment method used to build MSA; Bayesian calculation method; Best model evolutionary substitution.

### Calculating evolutionary conservation and mutation rate

MSA was used to determine the frequency (%) at which a given amino acid occurs at a given position within each histone protein across all species sampled. The somatic mutation count affecting each residue (histone protein and amino acid position) was calculated. Histone residues were grouped into mutation groups based on the number of cancer genomes harboring a mutation at that position (0, 1, 2, 3+ subjects). Mann–Whitney tests were used to determine differences in conservation frequency between two different mutation groups (e.g., residues with 0 vs. 3+ mutations).

### DepMap cancer cell lines

DepMap cell line metadata and pre-processed WES mutation data in the form of variant call files (VCF) were downloaded from the DepMap Data Portal (https://depmap.org/portal/download/) for the version DepMap Public 21Q2^49^. Cell lines were filtered to exclude any non-cancerous cell lines (marked as ‘matched normal tissue’, ‘engineered’, ‘fibroblast’, ‘non-cancerous’, and ‘unknown origin’), from analyses. A total of 1703 cancer cell lines were included for analysis. Mutation data were filtered to exclude synonymous mutation calls and retain only non-synonymous alterations. Mutations were queried across histone-encoding genes. To filter out putative germline mutation events in the absence of patient-matched germline DNA, any mutations present at ≥0.01 maximum population allelic frequency according to gnomAD were excluded.

### DepMap drug sensitivity analysis

Drug response data from the PRISM Repurposing 19Q4 primary drug screen^50^ were downloaded from the DepMap database. Drug response data represented results of primary drug library screen, the readout being cell barcode abundance in lysed cells. Results files were downloaded directly from the DepMap data portal and consisted of replicate collapsed log-fold change values of drug-treated, relative to dimethylsulfoxide (DMSO) control treated, cells. Cell lines marked as ‘FAILED-STR’ (*n* = 10; failed short tandem repeat analysis for cell line identity) were removed from analyses, and non-cancer cell lines were excluded. Only cancer cell lines with annotated histone mutation status, from the 21Q2 CCLE mutation profiling data (see above), were included for analysis.

Differences in drug response between histone mutation subtypes were identified in a preliminary ‘discovery’ analysis to identify candidate drugs for further investigation. For each drug included in the PRISM screen, we determined normality of drug response data distribution within each comparison group (histone mutant vs. histone WT) using Shapiro-Wilk’s tests. For normally distributed drug responses (Shapiro test, *p* > 0.05 for both comparison groups), unpaired t-tests were used with Welch’s correction to account for unequal variances between comparison groups. If either/both comparison groups exhibited non-parametric drug responses (Shapiro test, p < 0.05), unpaired two-sample Wilcoxon rank sum tests were used. Subsequently, Benjamini–Hochberg correction was applied to control the false discovery rate (FDR) by correcting *p*-values. Drugs with lowest FDR in the preliminary screen were selected for further analyses.

Subsequently, drug responses for candidate drugs (based on lowest FDR values from the preliminary discovery screen) were selected for manual comparison between histone mutant subtypes using t-tests (normally distributed drug response data) or Wilcoxon rank sum tests (non-normally distributed) to test the null hypothesis that there were no differences in drug response between histone mutation subtypes. Linear models were used to identify features that predicted drug response, including test variables (histone mutation subtype) and clinical co-variates (sex, age, and cancer type of the donor patient).

### Survival analyses

Kaplan–Meier progression free and overall survival curves were generated using GraphPad Prism 9 software. *P*-values were obtained from log-rank (Mantel–Cox) tests.

### Other statistical analyses

To test for differences in histone mutation rates between different age groups and glioma grades among the CNS tumor cohort, Chi-square tests were used where sample sizes for each comparison group were >5, and Fisher’s exact tests were used to derive two-sided *p*-values where sample sizes were ≤5. Bonferroni multiple testing correction was performed to adjust *p*-values in cases where multiple comparisons were made. To compare age at diagnosis between histone mutant subtypes, Mann–Whitney tests were performed between two comparison groups. Multiple testing correction was performed using Bonferroni correction to manually adjust *p*-values (by multiplying the *p* value by the number of tests performed, *n* = 3). Analyses were performed using GraphPad Prism 9 software. All statistical analyses were reviewed by a biostatistician (HGD).

### Reporting summary

Further information on research design is available in the Nature Research Reporting Summary linked to this article.

### Supplementary information

Supplemental Material

Supplementary Data File 1

Reporting Summary

## Data Availability

No new datasets were generated in this study. The TCGA Pan-Cancer Atlas public somatic mutation callset was obtained from https://gdc.cancer.gov/about-data/publications/pancanatlas (mc3.v0.2.8.PUBLIC.maf.gz). The ICGC PCAWG public somatic mutation callset was obtained from https://dcc.icgc.org/releases/PCAWG/consensus_snv_indel (final_consensus_passonly.snv_mnv_indel.icgc.public.maf.gz). The PBTA/Children’s Brain Tumor Network (CBTN) somatic mutation dataset was obtained from https://cavatica.sbgenomics.com/u/cavatica/openpbtadataset^24^.
