# Supplementary material for: Pan-cancer atlas of somatic core and linker histone mutations
Source: NPJ Genom Med. 2023 Aug 28;8:23. doi: 10.1038/s41525-023-00367-8 (PMC10462747; doi:10.1038/s41525-023-00367-8)
Supplement: Supplementary file 1 — Supplemental Material [file 41525_2023_367_MOESM1_ESM.pdf]

Supplementary Material

Supplementary Data File 1. Histone mutations in TCGA, ICGC PCAWG, and PBTA datasets. This table lists all histone gene mutations found among TCGA, ICGC PCAWG, and PBTA datasets that passed variant calling and filtering criteria (described in **Methods**). The unique patient identifier (ID), histone gene, and amino acid change are listed for each mutation.

ACC		
**Subtype**	**Core histone mutant**	**Core histone wildtype**
COC1	1	32
COC2	2	17
COC3	6	18
NA*	1	14
**Chi-Square**	6.466	
**df**	2	
**p-value**	0.0394	

UCS		
**Subtype**	**Core histone mutant**	**Core histone wildtype**
POLE	0	17
MSI	5	59
CN low	11	79
CN high	11	48
NA*	7	120
**Chi-Square**	5.949	
**df**	3	
**p-value**	0.1141	

Mesothelioma		
**Subtype**	**Linker histone mutant**	**Linker histone wildtype**
iCluster 1	2	18
iCluster 2	0	15
iCluster 3	0	19
iCluster 4	0	19
**Chi-Square**	5.449	
**df**	3	
**p-value**	0.1417	

Supplementary Data File 2. Chi-square analyses of histone mutation frequencies among ACC, UCS, and mesothelioma prognostic subtypes. This table lists the number of histone mutant subjects belonging to each previously defined prognostic subtype for ACC(*28*), UCS(*29*), and mesothelioma(*30*) cohorts from

the TCGA dataset. Chi-square tests were performed to compare histone mutation frequencies among ACC, UCS, and mesothelioma prognostic subtypes and the Chi-square statistics, degrees of freedom (df), and p-values are listed below the corresponding subject counts. *Subjects without available prognostic subtype data ('NA') were excluded from the Chi-square analyses.

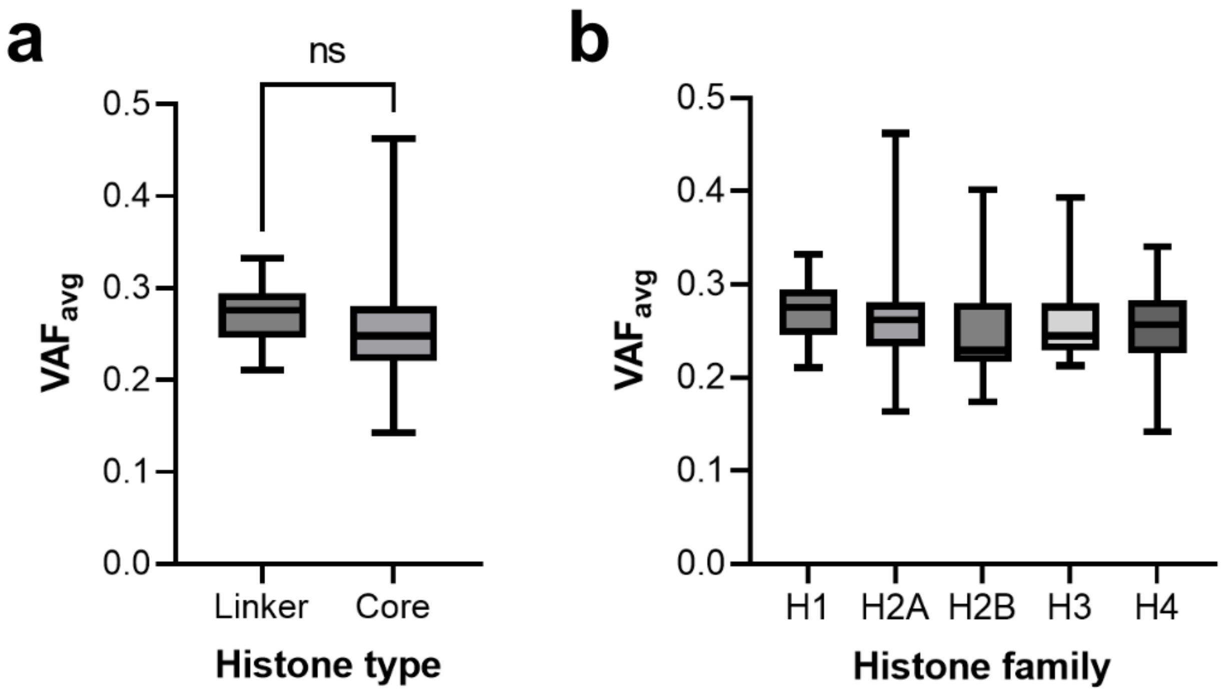

Supplementary Figure 1. Comparison of VAFs between histone families. (A) Box plots showing the VAF distributions of mutations affecting linker histone relative to core histone genes (Mann-Whitney test, $p>0.05$). **(B)** Box plots showing VAFs distributions for each histone family (H1, H2A, H2B, H3, H4). Upper and lower quartiles and medians are shown.

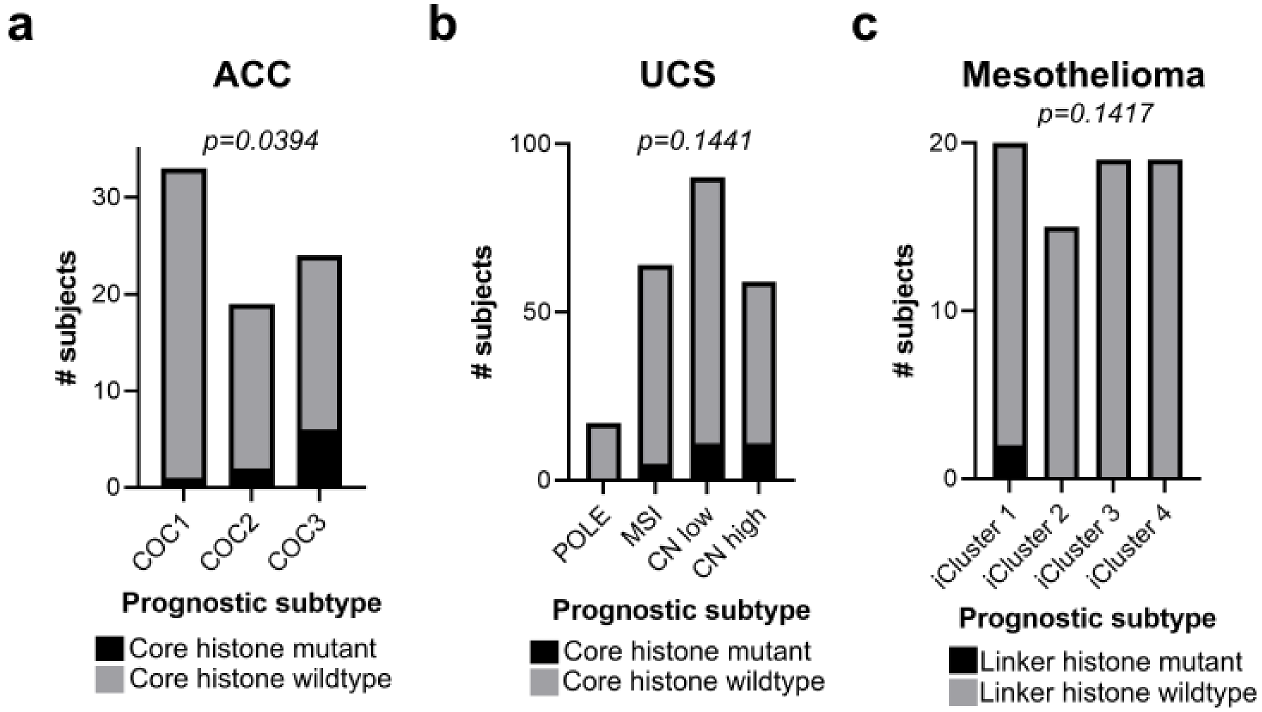

Supplementary Figure 2. Enrichment of histone mutations in prognostic molecular subtypes of ACC, UCS, and mesothelioma. Black bars show the number of subjects with mutant histone genes and grey bars show the number of subjects with wildtype histone genes. P values are based on Chi-square tests.

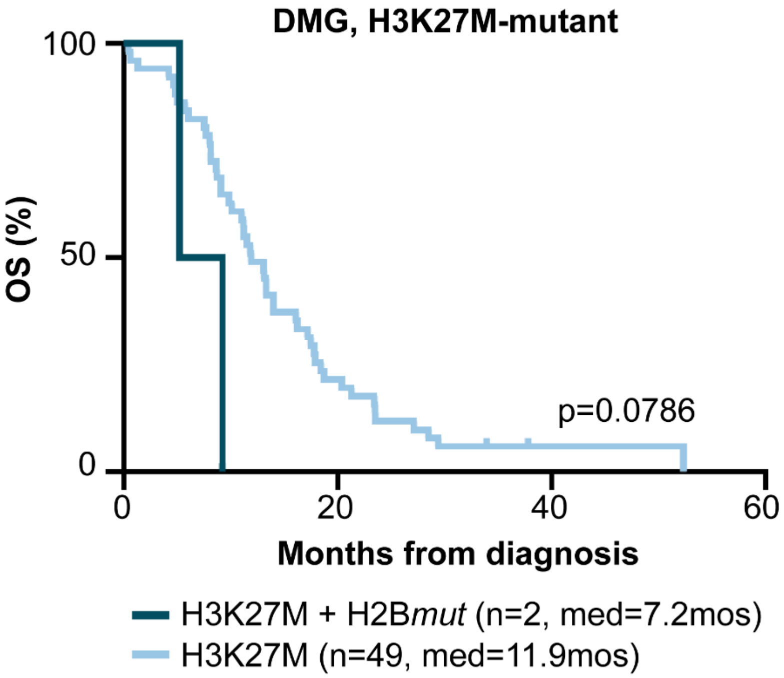

Supplementary Figure 3. Clinical implications of H2B mutations in DMG, H3 K27M mutant. Kaplan-Meier overall survival curve showing trend towards shorter survival outcomes in two DMG patients harboring co-occurring H3 K27M and H2B mutations, when compared to DMG harboring H3 K27M without additional histone gene alterations (n=49). P=0.0786, log-rank Mantel-Cox test.

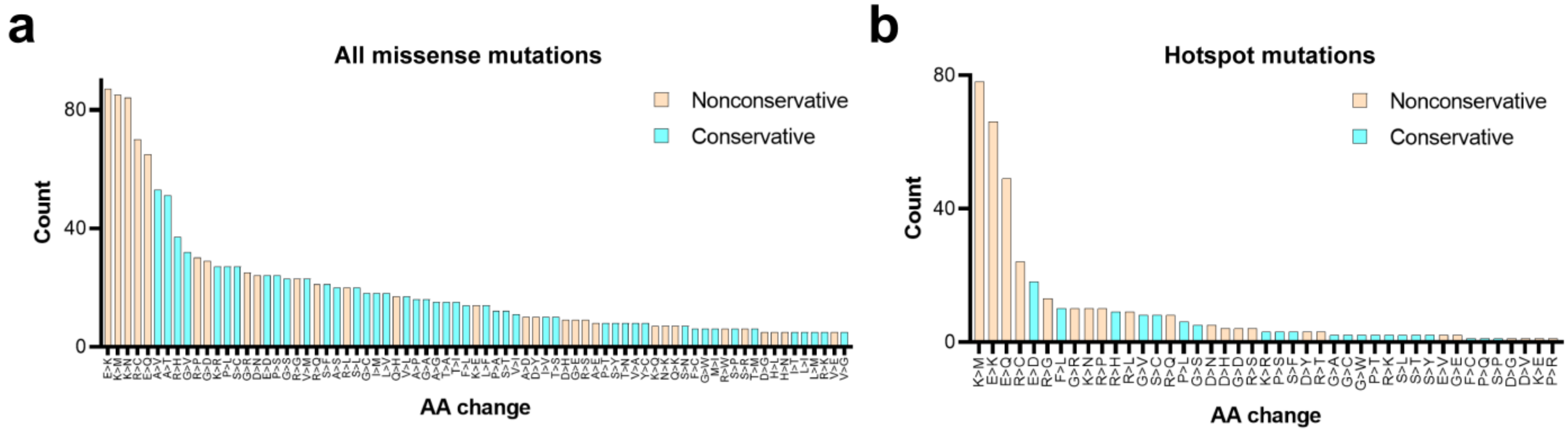

Supplementary Figure 4. Amino acid changes resulting from missense mutations to histone proteins. (A) Number of missense mutation events (count, y-axis) resulting in conservative vs. nonconservative substitutions. Cut-off at amino acid changes (AA change) with 5+ mutation counts. **(B)** Substitutions resulting from hotspot missense mutations.
