# Supplementary material for: Pan-cancer atlas of somatic core and linker histone mutations
Source: NPJ Genom Med. 2023 Aug 28;8:23. doi: 10.1038/s41525-023-00367-8 (PMC10462747; doi:10.1038/s41525-023-00367-8)
Supplement: Supplementary file 3 — Reporting Summary [file 41525_2023_367_MOESM3_ESM.pdf]

nature portfolio

Corresponding author(s): Sebastian Waszak, Javad Nazarian

Last updated by author(s): Jun 13, 2023

Reporting Summary

Nature Portfolio wishes to improve the reproducibility of the work that we publish. This form provides structure for consistency and transparency in reporting. For further information on Nature Portfolio policies, see our Editorial Policies and the Editorial Policy Checklist.

Statistics

For all statistical analyses, confirm that the following items are present in the figure legend, table legend, main text, or Methods section.

n/a	Confirmed	
☐	☒	The exact sample size (*n*) for each experimental group/condition, given as a discrete number and unit of measurement
☒	☐	A statement on whether measurements were taken from distinct samples or whether the same sample was measured repeatedly
☐	☒	The statistical test(s) used AND whether they are one- or two-sided<br>*Only common tests should be described solely by name; describe more complex techniques in the Methods section.*
☐	☒	A description of all covariates tested
☐	☒	A description of any assumptions or corrections, such as tests of normality and adjustment for multiple comparisons
☐	☒	A full description of the statistical parameters including central tendency (e.g. means) or other basic estimates (e.g. regression coefficient) AND variation (e.g. standard deviation) or associated estimates of uncertainty (e.g. confidence intervals)
☐	☒	For null hypothesis testing, the test statistic (e.g. *F*, *t*, *r*) with confidence intervals, effect sizes, degrees of freedom and *P* value noted<br>*Give P values as exact values whenever suitable.*
☒	☐	For Bayesian analysis, information on the choice of priors and Markov chain Monte Carlo settings
☒	☐	For hierarchical and complex designs, identification of the appropriate level for tests and full reporting of outcomes
☒	☐	Estimates of effect sizes (e.g. Cohen's *d*, Pearson's *r*), indicating how they were calculated

Our web collection on statistics for biologists contains articles on many of the points above.

Software and code

Policy information about availability of computer code

Data collection	All data in this study were downloaded from publicly available datasets (The Cancer Genome Atlas, Pan-Cancer Analysis of Whole Genomes, Pediatric Brain Tumor Atlas, and The Cancer Dependency Map datasets, as described and referenced in the Data Availability section of the Methods).
Data analysis	GraphPad Prism 9 and RStudio (version 2022.02.3+492) software were used to analyze data in this study.

For manuscripts utilizing custom algorithms or software that are central to the research but not yet described in published literature, software must be made available to editors and reviewers. We strongly encourage code deposition in a community repository (e.g. GitHub). See the Nature Portfolio guidelines for submitting code & software for further information.

Data

Policy information about availability of data

All manuscripts must include a data availability statement. This statement should provide the following information, where applicable:

- Accession codes, unique identifiers, or web links for publicly available datasets
- A description of any restrictions on data availability
- For clinical datasets or third party data, please ensure that the statement adheres to our policy

No new datasets were generated in the current study. The TCGA dataset was generated by the TCGA Research Network and is hosted on the National Cancer

Institute Genomic Data Commons Data Portal (https://www.cancer.gov/tcga). The PCAWG dataset is hosted on ICGC Data Portal(23) (https://dcc.icgc.org/releases/PCAWG). The CBTN dataset is hosted on the Kids First Data Resource Portal (DOI: 10.24370/SD_BHJXBDQ) (24).

Research involving human participants, their data, or biological material

Policy information about studies with human participants or human data. See also policy information about sex, gender (identity/presentation), and sexual orientation and race, ethnicity and racism.

Reporting on sex and gender	Biological sex was considered in the study to look for differences in histone gene mutation frequencies between male and female subjects. Biological sex information was obtained from publicly available clinical data on patients from The Cancer Genome Atlas, Pan-Cancer Analysis of Whole Genomes, Pediatric Brain Tumor Atlas, and Cancer Dependency Map datasets.
Reporting on race, ethnicity, or other socially relevant groupings	N/A
Population characteristics	Human subjects from publicly available datasets (The Cancer Genome Atlas, Pan-Cancer Analysis of Whole Genomes, Pediatric Brain Tumor Atlas, and Cancer Dependency Map) were included in this study and consisted of pediatric, adolescent, and adult subjects diagnosed with cancer. More than thirty cancer types including both solid and hematological cancers were included in the study.
Recruitment	N/A, publicly available data were used and no subjects were recruited for this study.
Ethics oversight	Children's National Hospital

Note that full information on the approval of the study protocol must also be provided in the manuscript.

Field-specific reporting

Please select the one below that is the best fit for your research. If you are not sure, read the appropriate sections before making your selection.

☒ Life sciences ☐ Behavioural & social sciences ☐ Ecological, evolutionary & environmental sciences

For a reference copy of the document with all sections, see nature.com/documents/nr-reporting-summary-flat.pdf

Life sciences study design

All studies must disclose on these points even when the disclosure is negative.

Sample size	No sample size calculations were performed, instead the authors amassed publicly available cancer genome data from a large number (>12,700) of subjects to create a comprehensive map of histone gene mutations across cancers
Data exclusions	No data were excluded from the study
Replication	Findings were not replicated or reproduced since the study was focused on analyzing publicly available cancer genome data to define mutation patterns across cancer types, thus experimental replication was not applicable
Randomization	No experimental groups were used or tested in this study
Blinding	Blinding was not relevant to this study as there were no experimental or control groups being tested

Reporting for specific materials, systems and methods

We require information from authors about some types of materials, experimental systems and methods used in many studies. Here, indicate whether each material, system or method listed is relevant to your study. If you are not sure if a list item applies to your research, read the appropriate section before selecting a response.

Materials & experimental systems

n/a	Involved in the study
☒	☐ Antibodies
☒	☐ Eukaryotic cell lines
☒	☐ Palaeontology and archaeology
☒	☐ Animals and other organisms
☒	☐ Clinical data
☒	☐ Dual use research of concern
☒	☐ Plants

Methods

n/a	Involved in the study
☒	☐ ChIP-seq
☒	☐ Flow cytometry
☒	☐ MRI-based neuroimaging
